# Fine-mapping studies distinguish genetic risks for childhood- and adult-onset asthma in the HLA region

**DOI:** 10.1186/s13073-022-01058-2

**Published:** 2022-05-24

**Authors:** Selene M. Clay, Nathan Schoettler, Andrew M. Goldstein, Peter Carbonetto, Matthew Dapas, Matthew C. Altman, Mario G. Rosasco, James E. Gern, Daniel J. Jackson, Hae Kyung Im, Matthew Stephens, Dan L. Nicolae, Carole Ober

**Affiliations:** 1 Department of Human Genetics, University of Chicago, Chicago, IL 60637 USA; 2 Section of Pulmonary and Critical Care, Department of Medicine, University of Chicago, Chicago, IL 60637 USA; 3 Department of Statistics, University of Chicago, Chicago, IL 60637 USA; 4 Division of Allergy and Infectious Diseases, Department of Medicine, University of Washington, Seattle, WA 98109 USA; 5 Systems Immunology Program, Benaroya Research Institute, Seattle, WA 98101 USA; 6 Department of Pediatrics, University of Wisconsin, School of Medicine and Public Health, Madison, WI 53706 USA; 7 Section of Genetic Medicine, Department of Medicine, University of Chicago, Chicago, IL 60637 USA

**Keywords:** Asthma, HLA, Fine-mapping

## Abstract

**Background:**

Genome-wide association studies of asthma have revealed robust associations with variation across the human leukocyte antigen (HLA) complex with independent associations in the HLA class I and class II regions for both childhood-onset asthma (COA) and adult-onset asthma (AOA). However, the specific variants and genes contributing to risk are unknown.

**Methods:**

We used Bayesian approaches to perform genetic fine-mapping for COA and AOA (*n*=9432 and 21,556, respectively; *n*=318,167 shared controls) in White British individuals from the UK Biobank and to perform expression quantitative trait locus (eQTL) fine-mapping in immune (lymphoblastoid cell lines, *n*=398; peripheral blood mononuclear cells, *n*=132) and airway (nasal epithelial cells, *n*=188) cells from ethnically diverse individuals. We also examined putatively causal protein coding variation from protein crystal structures and conducted replication studies in independent multi-ethnic cohorts from the UK Biobank (COA *n*=1686; AOA *n*=3666; controls *n*=56,063).

**Results:**

Genetic fine-mapping revealed both shared and distinct causal variation between COA and AOA in the class I region but only distinct causal variation in the class II region. Both gene expression levels and amino acid variation contributed to risk. Our results from eQTL fine-mapping and amino acid visualization suggested that the HLA-DQA1*03:01 allele and variation associated with expression of the nonclassical *HLA-DQA2* and *HLA-DQB2* genes accounted entirely for the most significant association with AOA in GWAS. Our studies also suggested a potentially prominent role for HLA-C protein coding variation in the class I region in COA. We replicated putatively causal variant associations in a multi-ethnic cohort.

**Conclusions:**

We highlight roles for both gene expression and protein coding variation in asthma risk and identified putatively causal variation and genes in the HLA region. A convergence of genomic, transcriptional, and protein coding evidence implicates the *HLA-DQA2* and *HLA-DQB2* genes and HLA-DQA1*03:01 allele in AOA.

**Supplementary Information:**

The online version contains supplementary material available at 10.1186/s13073-022-01058-2.

## Background

Asthma is a chronic, inflammatory disease of the airways, affecting over 330 million people worldwide and representing a significant global health burden [1]. Genome-wide association studies (GWASs) have reported over 150 independent loci associated with asthma [2–6], including highly replicated, significant associations at the human leukocyte antigen (HLA) region on chromosome 6p21. Recently, we performed a GWAS for childhood-onset asthma (COA) and adult-onset asthma (AOA) [3] in individuals from the UK Biobank [7]. Each revealed independent, broad regions of association spanning HLA class I (*HLA-C*/*B*) and class II (*HLA-DR*/*DQ*) genes. While the HLA class II region was the most significant locus for AOA, variants in this region had greater effect sizes for COA compared to AOA [3]. In contrast, associations with variants in the class I region were similar and had similar effect sizes in both.

Overall, the HLA region is the most frequently associated locus with asthma and allergic diseases [8]. Whereas its central role in adaptive immunity has been extensively characterized [9–11], determining the causal variants and their putative functions has been particularly challenging due to the remarkably high gene density, extraordinary levels of genetic polymorphism, and striking linkage disequilibrium (LD) that characterize this region [12, 13]. These features make identification of causal disease-associated variants and the genes that underlie these associations particularly challenging. As a result, the specific HLA region variants and genes contributing to asthma risk are not known.

In this study, we hypothesized that the causal variants at the HLA locus include those that are both shared and distinct to COA and AOA and that some causal variants exert their effects on asthma risk by modifying the expression of HLA genes while others alter protein properties by changing amino acid sequences in functional domains. Using Bayesian approaches for fine-mapping GWAS loci (genetic fine-mapping) and for fine-mapping expression quantitative trait loci (eQTLs; expression fine-mapping) of HLA region genes in cell types relevant to asthma, we identified putatively causal COA and AOA variants and genes in the HLA class I and class II regions and replicated a subset of causal variants in an ethnically diverse sample.

## Methods

### Study subjects in the discovery and replication samples

We examined COA and AOA HLA loci in a discovery cohort comprised of the same adult individuals from the UK Biobank data release July 19, 2017, and using the same inclusion/exclusion criteria and phenotype definitions reported in Pividori et al*.* [3]. Briefly, we filtered out individuals with poor-quality genotypes, ambiguous sex assignments, and related individuals [3]. Subjects included in the discovery analysis were restricted to White British ancestry (UK Biobank Data-Field 22006). COA cases were subjects with self-reported doctor-diagnosed asthma before 12 years of age (*n*=9432), AOA cases were subjects with self-reported doctor-diagnosed asthma between 26–65 years of age (*n*=21,556), and controls were subjects with no reported asthma up to the latest age of study participation (*n*=318,167). Individuals with chronic obstructive pulmonary disease, chronic bronchitis, and emphysema were excluded from the AOA and control groups.

The replication cohort consisted of individuals from the UK Biobank who were not in the White British ancestry set. We used the same definitions as above for COA (*n*=1686), AOA (n=3666), and non-asthmatic controls in the replication cohort (*n*=56,063). The self-reported ancestry for the replication cohort is 70.7% White, 12.4% Black or Black British, and 16.8% Asian or Asian British (Additional file 1: Fig. S1).

### Genotypes and HLA alleles

Allele dosages were extracted for genotyped and imputed SNPs from UK Biobank v3 within the boundaries of the asthma HLA loci in the COA and AOA GWAS as defined by Pividori et al*.* [3] using the rbgen 0.1 package in R 3.6.1 [14]. All SNPs that passed the following genotype quality control filters: call rate > 95% or information score > 80%, Hardy-Weinberg equilibrium test *p*-value > 1 × 10^–10^, and minor allele frequency (MAF) > 0.1% were included, as previously described [3]. A total of 8624 and 10,006 SNPs passed the genotype filters at the HLA class I and class II locus, respectively. Four-digit resolution (also known as two-field resolution) of classical HLA allele dosages were imputed from SNP data by the UK Biobank using HLA*IMP:02 [7, 15]. We excluded alleles with low frequencies (<1%, specific to each self-reported race). We translated the imputed HLA allele dosages to their corresponding amino acid dosages using publicly available data from SNP2HLA [16] (http://software.broadinstitute.org/mpg/snp2hla/) that map HLA alleles to their amino acid sequences. All amino acid polymorphisms were encoded as biallelic, as previously described [16–18]. Amino acid polymorphisms with low frequencies (<1%) were excluded. Base-pair positions for variants, genes, and other genomic features are based on Human Genome Assembly hg19 [19, 20]. We use the term “HLA allele” when referring to the four-digit nomenclature, “amino acid polymorphism” when the amino acid is the target of analysis, and “SNP” when the SNP is the target of analysis. We note that SNPs determine amino acid polymorphisms and amino acid polymorphisms determine HLA alleles.

### Variant associations

Associations between variants (SNPs, HLA alleles, and amino acid polymorphisms) and COA and AOA were tested using logistic regression. Sex and the first 10 ancestral principal components (PCs) were included as covariates in the analyses in White British subjects. We used the test for heterogeneity from METAL [21] to determine if variant effects were shared between COA and AOA. We focused on significantly associated variants and amino acid polymorphisms and applied a Bonferroni correction to the results from the heterogeneity test*.*

To determine whether the associations with variants of interest were driven in part by allergy, we extracted allergy status (any eczema, hay fever, and/or food allergy) and performed regressions where we (1) included allergy status as a covariate in the regression, (2) used an interaction term between allergy status and the variant of interest, and (3) examined associations excluding all individuals with allergy. To determine whether there were any sex differences in HLA-associated risks, we (1) performed associations for the candidate variants separately in males and females and (2) include an interaction term between sex and the variant in the regression model.

### Fine-mapping the HLA region

Sum of Single Effects (SuSiE) [22] (susieR R package version 0.9.0) was used to fine-map the asthma-associated HLA loci and determine putatively causal variants for COA and AOA, separately in the class I and class II regions. SuSiE is a Bayesian analog of stepwise conditional analysis that improves on previous methods by taking account of the uncertainty of the selection of associated SNPs. Additionally, SuSiE can handle individual-level data (allowing us to examine SNPs, HLA alleles, and amino acid polymorphisms), detect multiple causal signals, and identify causes when the variable with the lowest *p*-value is not the causal variable [22]. SuSiE reports credible sets, which are sets of variants that include at least one causal variant with high probability. To assess whether SNPs, amino acid polymorphisms, and/or HLA alleles were causal for asthma risk, genotype dosages for these three types of polymorphisms were included in a single genotype matrix: class I SNPs, HLA alleles, and HLA amino acid polymorphisms were examined together for the fine-mapping studies in the class I region; class II SNPs, HLA alleles, and HLA amino acid polymorphisms were examined together for the class II region. See Additional file 1: Supplementary Methods for more details.

### Gene expression and eQTL studies

To test whether SNPs identified in the fine-mapping results using SuSiE are eQTLs in asthma-relevant cell types, RNA-seq data from three cell sources from ethnically diverse individuals was evaluated (Additional file 1: Table S1). Lymphoblastoid cell lines (LCLs), peripheral blood mononuclear cells (PBMCs), and upper airway (nasal) epithelial cells (NECs) were considered as surrogates for immune, lung, and epithelial tissues that were implicated as relevant cells/tissues in asthma risk in the COA and AOA GWAS [3]. The LCLs were derived from blood previously collected from 398 Hutterites [23], a founder population of European descent. Unstimulated PBMCs were derived from blood (*n*=132) [24] and NECs from nasal swabs (*n*=188) [25], which were previously collected from children from the URban Environment and Childhood Asthma (URECA) birth cohort [26]. In both studies, written informed consent was obtained from the parents of children, and assent from children age 6 or older. Both studies were approved by institutional review boards, and all study subjects were assigned randomly generated ID codes.

RNA-sequencing reads from these studies were remapped. For the polymorphic HLA genes, we aligned RNA-seq reads to reference sequences from the International ImMunoGeneTics (IMGT) database [27] for each individual’s known HLA type [28] (see Additional file 1: Supplementary Methods for more details). For eQTL mapping in the PBMCs and NECs, we performed linear regressions with QTLtools [29] using a nominal pass and *cis-*window size of ±1 Mb from the transcription start site (TSS). We performed eQTL mapping in the LCLs using Genome-wide Efficient Mixed Model Association (GEMMA) [30], including a kinship matrix as a random effect to account for relatedness between the Hutterites. Relevant covariates were included for all tests (see Additional file 1: Supplementary Methods for additional details). In the LCLs, some SNPs in the AOA or COA credible sets had high missingness. In these instances, we ran the eQTL mapping in the subset of individuals with high-quality genotypes. In the PBMCs and NECs, some SNPs in the AOA or COA credible sets failed QC in genotypes extracted from whole genome sequence data. In those instances, we extracted high-quality genotypes from the Illumina MEGA array in the subset of individuals that had previous array-based genotyping and used those genotypes in the eQTL studies.

### Fine-mapping eQTLs

We performed eQTL fine-mapping in three asthma-relevant cell types, LCLs, PBMCs, and NECs, using SuSiE for the expression of genes in which SNPs in any of the COA or AOA credible sets were significantly associated with their expression at a false discovery rate (FDR) [31] threshold of 0.05. The same covariates in each of the eQTL studies were regressed from the dataset (see Additional file 1: Supplementary Methods).

SuSiE was used to fine-map eQTLs using a window of ± 1 Mb around the TSS of each gene. SNPs in any of the eQTL credible sets were then compared to the SNPs in the COA and AOA GWAS credible sets to assess overlap.

### Structural visualization of amino acid variants

Based on results of fine-mapping, visualization of amino acid polymorphisms was performed for each associated coding variation (amino acid polymorphism). Amino acid polymorphisms were aligned to their positions on the protein. Crystal X-ray structures were downloaded from the Protein Data Bank (PDB) [32] for HLA molecules containing the risk/protective amino acid variants of interest, if available: 5VGE (HLA-C*07:02) [33], 6DIG (HLA-DQA1*01:02/HLA-DQB1*06:02) [34], and 4D8P (HLA-DQA1*03:01/HLA-DQB1*02:01) [35]. The locations of amino acids within the HLA molecules were visualized with PyMOL v2.0.7 [36] (https://pymol.org/2/).

### Replication of fine-mapping results

Because variants within a credible set are highly correlated, we selected candidate variants (tag SNPs) from each credible set for replication. These included rs2428494 (the shared class I COA and AOA credible set 1 [CS1] SNP), HLA-C p.11 (class I COA credible set 2 [CS2] amino acid polymorphism), rs28407950 (class II COA CS1 lead SNP), rs35571244 (lead class II COA CS2 SNP), rs9272346, rs1063355, rs3828789, and rs9274660 (class II AOA CS1 SNPs that were also in the eQTL CSs and overlapped with LCL enhancer marks), and HLA-DQA1*03:01 (class II AOA CS2 HLA allele). We performed logistic regressions for COA and AOA separately in self-reported White (excluded from White British Ancestry cohort), Asian British, and Black British individuals from the UK Biobank and then performed a meta-analysis as implemented in METAL [21]. Logistic regressions were performed using sex and the top 20 ancestry PCs as covariates in a one-sided test that required the direction of effect to be the same as in the discovery sample.

## Results

### HLA allele and amino acid associations

Similar to our previous study of White British individuals [3], we identified 9432 COA cases, 21,556 AOA cases, and 318,167 shared non-asthma controls (Table 1). Because our previous GWASs did not include HLA alleles or amino acid variants [3], we first considered the imputed 4-digit HLA alleles [37] provided by the UK Biobank and identified a total of 78 alleles for the *HLA-A* (*n*=13), *HLA-B* (*n*=18), *HLA-C* (*n*=14), *HLA-DRB1* (*n*=15), *HLA-DQB1* (*n*=12), and *HLA-DQA1* (*n*=7) genes that met the frequency threshold of 0.01. Of the 78 HLA alleles at six loci, 19 were associated with COA and 14 with AOA at a genome-wide significance threshold (*p*<5 × 10^−8^; Additional file 2: Table S2). Overall, the effect sizes of associated alleles were larger for COA compared to AOA (Additional file 1: Fig. S2). Our results were concordant using either an additive or dominant model (Additional file 2: Table S3). The only alleles that showed significant heterogeneity were HLA-DQA1*01:02, HLA-DQA1*03:01, HLA-DQB1*03:02, HLA-DQB1*06:02, and HLA-DRB1*15:01 (Additional file 2: Table S4).

**Table 1:** Sample composition

	Childhood-onset asthma	Adult-onset asthma	Controls
Sample size	9432	21,554	318,167
Mean age of asthma onset in years (SD)	6 (3)	44 (10)	NA
Any asthma medication use (%)	41.6	44.8	0.5
Female sex (%)	40.7	63.6	53.5
Allergic disease (ever, %)	34.0	24.6	10.6
Allergic rhinitis (%)	26.9	21.6	8.6
Atopic dermatitis (%)	11.9	4.3	2.3
Food allergy (%)	1.5	0.8	0.4
Mean FEV1 percent predicted (SD)	87.62 (18.20)	91.80 (17.05)	98.41 (15.62)
Mean FEV1/FVC (SD)	0.71 (0.08)	0.74 (0.07)	0.76 (0.06)
Mean eosinophil count (SD)	0.23 (0.18)	0.22 (0.17)	0.17 (0.12)
Mean tobacco exposure in home (SD)^a^	0.58 (4.89)	0.62 (4.93)	0.51 (4.35)
Current tobacco smoking (% never/occasionally/most days)	91.4/ 6.0/ 2.6	93.5/ 4.7/ 1.8	90.4/ 7.0/ 2.6

^a^Hours/week among non-smokers

*SD* standard deviation

After filtering out low-frequency amino acid polymorphisms (<1%), we tested the 741 amino acid polymorphisms at the six HLA loci for associations with COA and AOA. Of these, 188 amino acid polymorphisms were associated with COA and 152 were associated with AOA (*p*<5 × 10^−8^, Additional file 2: Table S5). *P*-values were overall more significant, and estimated ORs were larger for class II compared to class I HLA alleles and amino acid polymorphisms (Additional file 1: Fig. S3). The magnitude of the ORs was also generally larger for COA compared to AOA. Both of these observations are consistent with the GWAS results [3]. Using a test for heterogeneity, eight *HLA-DQA1*, seven *HLA-DQB1*, and 11 *HLA-DRB1* amino acid polymorphisms were significant, suggesting age of onset-specific effects (Additional file 2: Table S4).

### Fine-mapping the HLA class I region

To fully capture genetic variation at the HLA region, we combined the genotypes for 19,499 HLA alleles, amino acid polymorphisms and SNPs, and performed genetic fine-mapping on the combined set of variants separately in the class I and class II regions, with the goal of identifying causal variation for COA and AOA. We used the Bayesian regression method SuSiE [22] to perform fine-mapping of the 9021 combined variants at the class I locus using the locus boundaries defined previously [3].

**Fig. 1:**
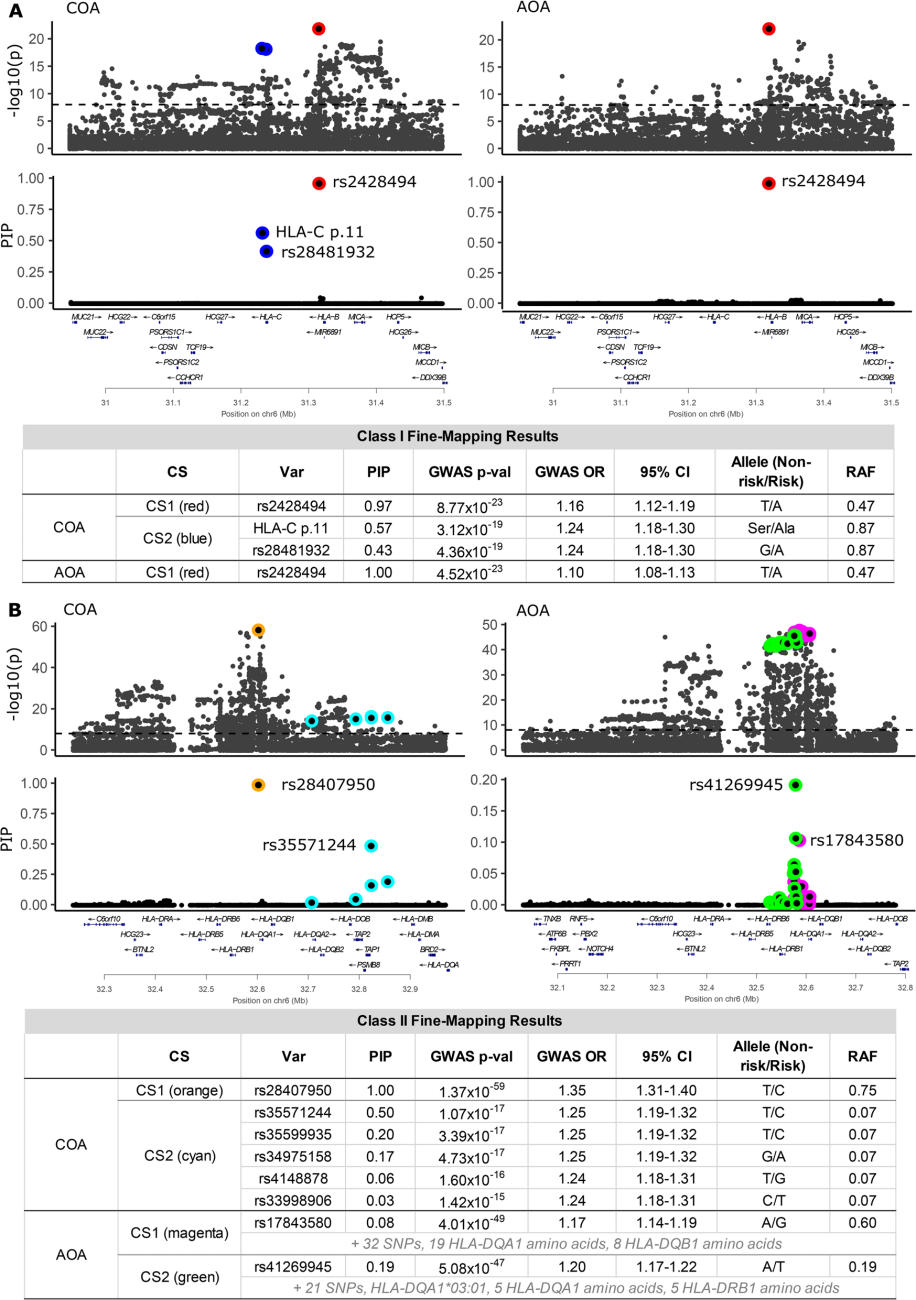
Fine-mapping results for childhood- and adult-onset asthma. In the **A** HLA class I and **B** HLA class II region, the upper panels show the −log_10_(*p*-values) from the GWAS [3]; the dashed line indicates genome-wide significance. The lower panels show the fine-mapping probabilities (PIPs) for the same variants. The colors represent the level-95% credible sets (HLA class I: CS1=red, CS2=blue; HLA class II CS1=orange and CS2=cyan for COA, and CS1=magenta, CS2=green for AOA). The tables show summaries of the variants in each credible set. A total of 9021 variants (SNPs, HLA alleles, amino acid polymorphisms) were examined in the class I region and 10,428 variants in the class II region

Fine-mapping the COA class I locus identified two credible sets, indicating the presence of two independent associations with COA in the class I region. Credible set 1 (CS1) consisted of a single variant and CS2 consisted of two variants. The CS1 SNP (rs2428494) (red point, Fig. 1A) is in an intron of *HLA-B*, and its posterior inclusion probability (PIP) is 0.97. This was the lead SNP in both the COA and AOA GWASs at the HLA class I locus [3]. In CS2 (blue points, Fig. 1A), the probability was nearly equally divided between two highly correlated variants (LD *r*^2^ = 0.99, calculated from our data), with PIP values of 0.43 and 0.57 for rs28481932 and HLA-C p.11, respectively. SNP rs28481932 is upstream of *HLA-C*, and HLA-C p.11 is an amino acid polymorphism in HLA-C (p.11 Ala/Ser). The risk amino acid (alanine) is on HLA-C*02, *03, *05, *06, *07, *08, *12, *15, *16, *17, and *18 alleles in this sample (including rare alleles), and the amino acid (serine) that is associated with decreased risk of asthma is on HLA-C*01:02, *04:01, *04:07, *14:02, and *14:03 alleles. The HLA class I locus in AOA included one credible set with rs2428494 having a PIP of 1.00 (Fig. 1A). Therefore, in the class I region, rs2428494 is a shared causal SNP for COA and AOA and HLA-C p.11 or rs28481932 is a causal variant for COA. Alternatively, one or both may tag untyped or rare causal variation in LD with the candidate variants identified by SuSiE.

The odds ratios (ORs) remained largely unchanged when accounting for allergy in the associations, and no sex differences in risk were observed for any of these variants, suggesting that the associations were not reflecting confounding due to the presence of allergic diseases in either COA or AOA (Additional file 1: Table S6, S7).

### Fine-mapping the HLA class II region

We used 10,428 combined variants at the class II region for fine-mapping. Two credible sets were identified for COA (Fig. 1B). CS1 (orange point) included one SNP (rs28407950; PIP 1.00) located downstream of *HLA-DQB1*. This SNP was the lead GWAS SNP in the HLA class II region for COA [3]. CS2 (cyan points) contained five SNPs spanning 152 kb. The SNP (rs35571244) with the highest PIP (0.50) was located at the proximal end of the class II region upstream of *TAP1*. The minimum *r*^2^ between all variants in CS2 was 0.79 (median *r*^2^=0.99). No HLA alleles or amino acid polymorphisms were included in either of the COA credible sets.

Two credible sets were also identified for AOA, but neither included variants in the COA credible sets (Fig. 1B, Additional file 2: Table S8). CS1 (magenta points) contained 60 variants: 33 SNPs, 19 *HLA-DQA1* amino acid polymorphisms, and eight *HLA-DQB1* amino acid polymorphisms. The minimum *r*^2^ between all variants in CS1 was 0.94 (median *r*^2^=0.99), spanning 32.1 kb across the *HLA-DQA1* and *HLA-DQB1* genes. The lead SNP in the AOA GWAS [3] (rs17843580), which is located downstream of *HLA-DQA1*, was also the lead SNP in CS1 (PIP 0.08), but because there were so many variants in this credible set all individual PIPs were small. CS2 (green) spanned 54.6 kb from *HLA-DRB5* to *HLA-DQA1*, and included 33 variants: 22 SNPs, five *HLA-DQA1* amino acid polymorphisms, one *HLA-DQA1* allele (HLA-DQA1*03:01), and five *HLA-DRB1* amino acid polymorphisms. The minimum *r*^2^ between all CS2 variants was 0.88 (median *r*^2^=0.96). The variant with the highest PIP was a SNP (rs41269945) located between *HLA-DQA1* and *HLA-DQB1*. Five perfectly correlated *HLA-DQA1* amino acids had the highest PIPs (each 0.07) among the amino acids: Thr26, Gln47, Arg56, Val76, and Thr187, which together define HLA-DQA1*03 alleles (*03:01, *03:02, and *03:03 in our data).

The association of these putatively causal SNPs with AOA were not due to confounding by inclusion of allergic diseases, as the results were similar when excluding individuals with these comorbidities (Additional file 1: Table S6). However, an interaction between the COA class II CS1 SNP (rs28407950) and sex was nominally significant (*p*=0.02) (Additional file 1: Table S7), reflecting a nominally larger effect in females (OR=1.43 95% CI [1.35–1.51]) compared to males (OR=1.31 95% CI [1.25–1.37]) for this variant.

### Fine-mapping simulations in the HLA region

Most previous fine-mapping studies excluded the HLA region due to its genomic complexities [38–40]. To validate that SuSiE accurately detects multiple independent causal signals in this region, we conducted simulations in each of the HLA class I and class II regions for both binary (e.g. case-control status) and quantitative (e.g. gene expression) outcomes (see Additional file 1: Supplementary Methods for additional details). In the null simulations (with zero causal variants), SuSiE accurately reported no credible sets (Additional file 1: Fig. S4). In the simulations ranging between one to three causal variants, SuSiE correctly detected the correct number of causal signals, each containing the true causal variant in the class I and class II regions for both the binary and quantitative outcomes. The true causal variant had the highest PIP 61% of the time. These simulations demonstrated that fine-mapping studies in the HLA region with SuSiE can accurately identify credible sets containing the true causal variants despite the complexities of this region.

### Fine-mapping eQTLs and functional annotations in the HLA region

Genetic variation can influence disease risk by altering protein function or by influencing expression levels of disease-associated genes. Earlier studies demonstrated different functional properties of HLA alleles defined by amino acid polymorphisms [9, 41], while more recent studies have shown regulatory effects of SNPs on HLA gene expression [42–46]. Our results suggested that both types of mechanisms may mediate the effects of HLA genes on asthma risk. We first assessed the potential regulatory effects of asthma-associated SNPs using gene expression data from three asthma-relevant cell types (Additional file 1: Table S1) from individuals with HLA types that were known or could be determined. This allowed us to map the RNA-sequencing (RNA-seq) data against sequences corresponding to each person’s known HLA type (Additional file 2: Table S9) and avoid mapping biases that arise from the large number of sequence differences between HLA alleles and reference genome (28).

**Fig. 2:**
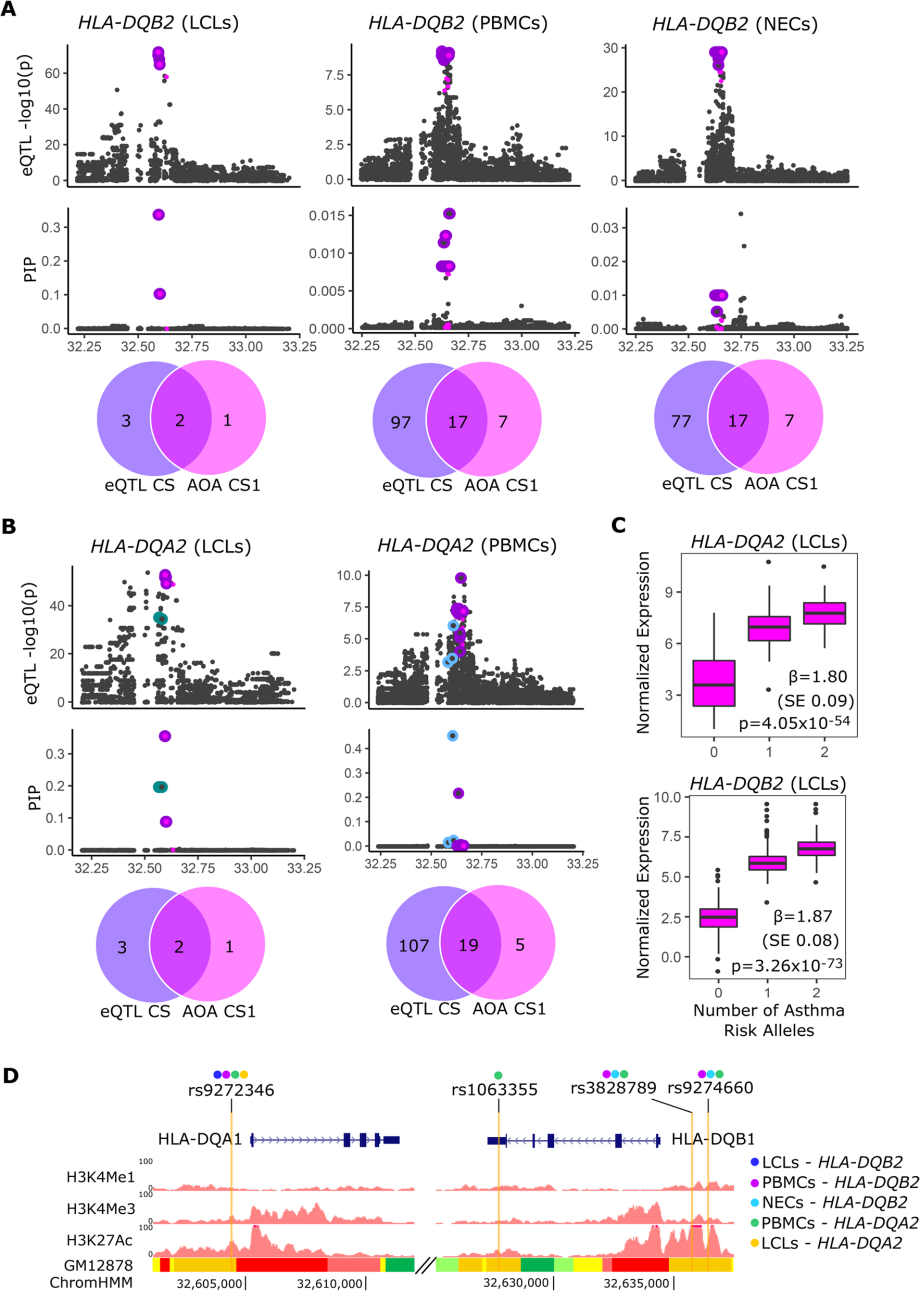
eQTL fine-mapping and functional annotations. For **A** *HLA-DQB2* and **B** *HLA-DQA2*, upper panels show eQTL −log_10_(*p*-values) and PIPs for each variant tested within ± 0.5 Mb of the gene TSS. The colored outlines show the eQTL credible sets; the magenta points are SNPs that were also in the class II AOA CS1. A purple outline around a magenta point shows the variants shared between the eQTL credible set and the class II AOA CS1. The green and blue outlines indicate other credible sets. *X*-axis position is in Mb (hg19). The Venn diagrams show the number of variants in the eQTL credible set and the number that overlaps with SNPs in the class II AOA CS1. **C** Normalized expression of *HLA-DQA2* (upper panel) and *HLA-DQB2* (lower-panel) in LCLs by the number of asthma-risk alleles (A) for rs9272346, a representative class II AOA CS1 SNP. **D** Four SNPs in the class II AOA CS1 and in one or more eQTL credible set overlapped with functional annotations. The colors above each rsID indicate which eQTL credible set they were in. All four SNPs overlapped with strong enhancers in GM12878 cells (LCLs; bottom track). The GM12878 ChromHMM results correspond to active promoters (red), weak promoters (light red), strong enhancers (orange), weak/poised enhancers (yellow), transcriptional transition/elongation (dark green), and weak transcription (light green). Modified from http://genome.ucsc.edu [47]

We first performed eQTL studies for all genes whose transcription start site (TSS) was ± 1 Mb of the SNPs in each credible set. This identified 245 significant eQTLs (false discovery rate, FDR<0.05) involving 46 SNPs and 17 genes (*CCHCR1*, *AL662844.4*, *MIR6891*, *PSBM9*, *TAP1*, *TAP2*, *PPT2*, *HLA-B*, *HLA-DQA1*, *HLA-DQB1*, *HLA-DQB1-AS1*, *HLA-DQA2*, *HLA-DQB2*, *HLA-DRB5*, *HLA-DRB6*, *HLA-DRB9*, and *HLA-DPB2*) (Additional file 2: Tables S10, S11). We then used SuSiE to perform fine-mapping for each gene with at least one eQTL in that cell type, resulting in expression fine-mapping of 15 genes in LCLs, three in PBMCs, and six in NECs (Additional file 1: Table S12). Five of the eQTL fine-mapping studies identified credible sets with SNPs that were also in the class II AOA CS1 (Additional file 2: Table S13). These included credible sets with eQTLs for *HLA-DQB2* in LCLs, PBMCs, and NECs (Fig. 2A) and *HLA-DQA2* in LCLs and PBMCs (Fig. 2B). The AOA CS1 risk alleles were associated with increased expression of both *HLA-DQA2* and *HLA-DQB2* in these cells (Fig. 2C, Additional file 1: Fig. S5). None of the other SNPs in eQTL fine-mapping credible sets overlapped with SNPs in the class II AOA CS2, the class II COA CS1 or CS2, or the AOA or COA class I credible sets.

To prioritize among the 20 SNPs that were in both the class II AOA CS1 and eQTL credible sets for *HLA-DQA2* or *HLA-DQB2*, we overlapped these SNPs with functional annotations from nine cell lines (GM12878 (LCLs), H1-hESC, K562, HepG2, HUVEC, HMEC, HSMM, NHEK, NHLF) available from ENCODE [48]. Four of the 20 SNPs overlapped an enhancer region in LCLs (Fig. 2D) and also resided in or near (approximately 70 to 700 bp) weak enhancer marks in three epithelial cell-derived lines (NHEK, keratinocytes; HMEC, mammary epithelial cells; HEPG2; liver hepatocellular cancer) (Additional file 1: Fig. S6). None of the SNPs overlapped with or were near enhancer marks in the other ENCODE cell lines. rs9272346, located upstream of *HLA-DQA1*, was in four of five of the eQTL credible sets. The other three SNPs were located near or within *HLA-DQB1*, with rs1063355, rs3828789, and rs9274660 in one, three, and three of the five eQTL credible sets, respectively. Surprisingly, the AOA GWAS lead SNP (rs17843580), which had the highest PIP in the AOA CS1, and the remaining 15 SNPs in CS1 did not overlap an enhancer region in any of the cell types (Additional file 1: Fig. S6).

### Structural visualization of amino acid variants

**Fig. 3:**
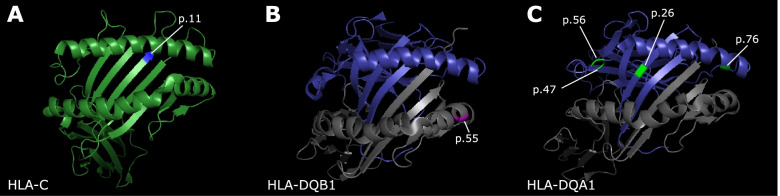
Localization of asthma-associated amino acid variants. Ribbon-figure representations of the peptide-binding pocket are shown for each HLA protein, and the amino acid variant in focus is highlighted. **A** HLA-C p.11, shown in blue, is located within the peptide-binding pocket of the HLA-C molecule [33] (forest green). **B** HLA-DQB1 p.55, shown in magenta, lies in the region that may interact with the TCR on the HLA-DQB1 [34] protein (gray) in complex with HLA-DQA1 (blue). **C** HLA-DQA1 p.26, p.47, p.56, and p.76 (green) shown on the HLA-DQA1 protein [35] (blue). p.26 lies in the peptide-binding pocket, p.76 in the region that may interact with the TCR, and p.56 and p.47 in regions outside of the peptide-binding pocket

HLA class I and class II molecules bind and present peptides to T cell receptors (TCRs). The polymorphic features of HLA class I and class II molecules, particularly in the peptide-binding domain, serve the crucial functions of diversifying antigen presentation and restricting pathogen-evasion of immune recognition. Therefore, we next explored the possibility that the amino acid variants with the highest PIPs in some of the credible sets affect peptide presentation or interactions with the TCR (Additional file 1: Table S14). The amino acid in the class I region with the highest PIP in the COA CS2, HLA-C p.11, is located within the peptide-binding pocket of the HLA-C protein (Fig. 3A). The serine allele was associated with protection from COA and is polar and uncharged; the alanine was associated with risk and is aliphatic and hydrophobic. Thus, both the location and structure of this polymorphic site suggest that peptide presentation by or other functional properties of HLA-C may be different between these alleles.

The amino acid with the highest PIP in the AOA class II CS1 was at position 55 in the HLA-DQB1 protein (Fig. 3B). Arginine contains a positively charged side chain and was associated with protection from AOA (*p*=4.50 × 10^−49^; OR=0.86, CI 0.84–0.88). This is a multiallelic position, with leucine and proline as alternate alleles; both were associated with asthma risk (Leucine: *p*=3.36 × 10^−6^; OR=1.06, CI 1.03–1.08; Proline: *p*=1.83 × 10^−28^; OR=1.12, CI 1.10–1.15). The risk variants were both hydrophobic whereas the protective variant was positively charged and polar. This location may be in a region that interacts with the TCR [49]. Because this variant is in strong LD with SNPs that are eQTLs in the class II AOA CS1 (median *r*^2^=0.99), it is unclear if this variant, the eQTLs, or both are causal for AOA.

Among the 10 amino acids in class II AOA CS2, HLA-DQA1 Ser26, Gln47, Arg56, and Val76 were in perfect LD with each other, had the highest PIPs of the amino acid polymorphisms in CS2, and were associated with AOA risk. These amino acids are present exclusively on the HLA-DQA1*03:01, 03:02, and 03:03 alleles (Additional file 2: Table S15). The HLA-DQA1*03:02 and HLA-DQA1*03:03 alleles occurred at low frequency (<1%) in this sample and were not included in the GWAS or fine-mapping studies. Of these four amino acids, Ser26 is in the peptide-binding pocket and Val76 is in a region that may interact with the TCR (Fig. 3C). The other amino acid polymorphisms were not in regions with obvious functional effects. At position 26, both the risk-associated serine and the protection-associated threonine have polar uncharged side chains, although the serine sidechain is smaller. Position 76 was multiallelic, with all three amino acids (valine, leucine, and methionine) having hydrophobic side chains, and valine having the smallest molecular weight. Position 187 was also perfectly correlated with these amino acids and captured in CS2 but was not part of the crystal structure. Our data support HLA-DQA1*03 alleles as risk alleles for AOA.

### Conditional analyses to assess independent effects

**Fig. 4:**
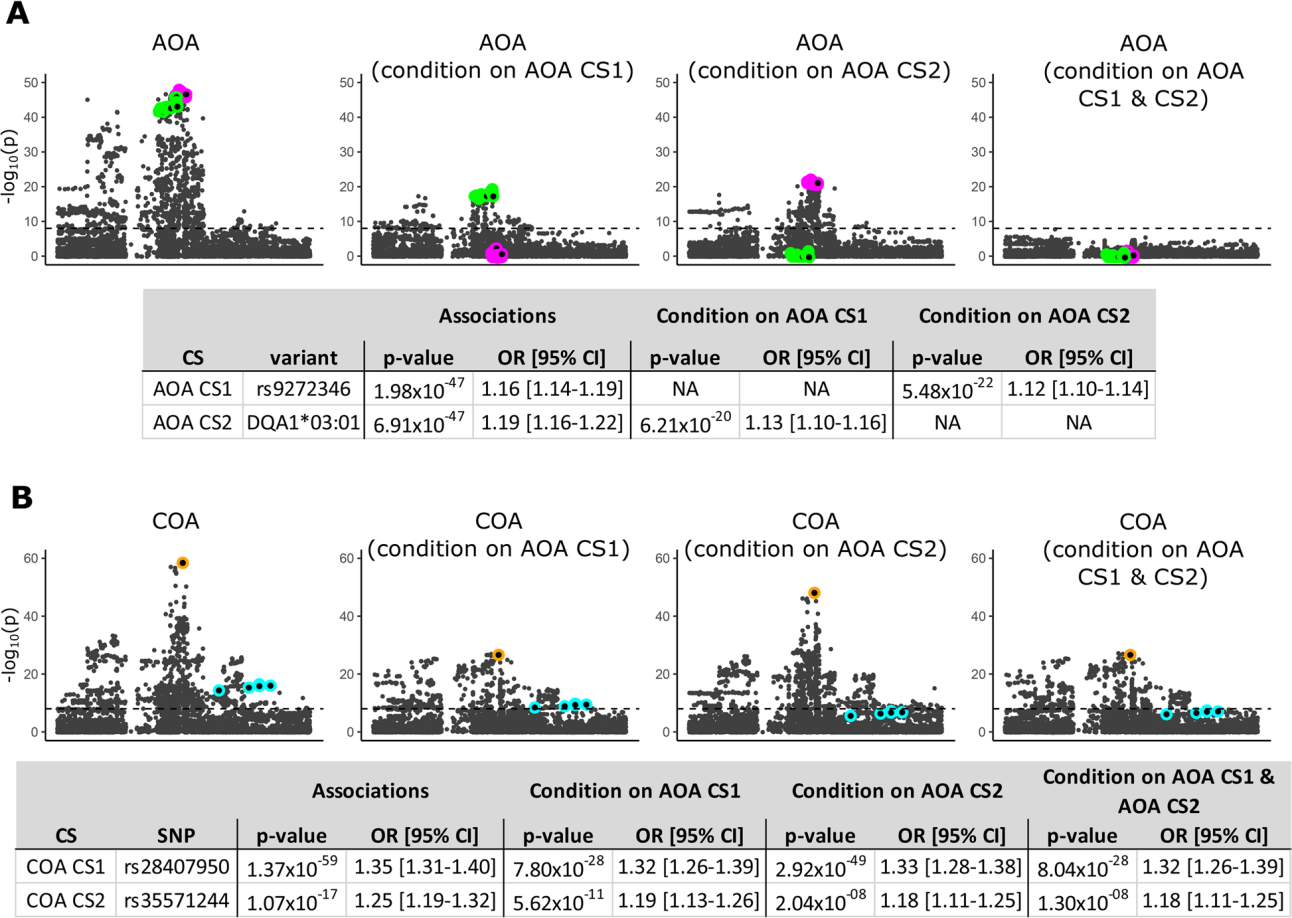
Class II conditional analyses. For **A** adult-onset asthma (AOA) and **B** childhood-onset asthma (COA), results show the −log_10_(*p*-value) for each variant after conditioning on rs9272346 (representative AOA CS1 SNP), and/or HLA-DQA1*03:01 (representative AOA CS2 variant). Colored outlines correspond to different credible sets; AOA CS1: magenta, AOA CS2: green, COA CS1: orange, and COA CS2: cyan. See Fig. 1 for comparison

To determine whether the candidate eQTL SNP for *HLA-DQA2* and *HLA-DQB2* (rs9272346) in CS1 and the HLA-DQA1*03:01 allele in CS2 accounts for all the association signal at the class II AOA locus, we performed three conditional analyses in which we included the number of alleles for rs9272346, for HLA-DQA1*03:01, and for both as covariates in association tests of variants at this locus with AOA (Fig. 4A). In the first conditional analysis including number of rs9272346 (CS1) alleles as a covariate, the AOA association signal for HLA-DQA1*03:01 remained genome-wide significant; in the second conditional analysis including the number of HLA-DQA1*03:01 alleles (CS2) as a covariate, the AOA association signal for rs9272346 also remained genome-wide significant. When both rs9272346 and HLA-DQA1*03:01 were included as covariates, the significance across the locus was reduced. These analyses indicate that the most significant asthma locus in AOA is due to variation in two credible sets, whose effects are likely attributable to their impact on expression of the *HLA-DQA2* and *HLA-DQB2* genes and of the HLA-DQA1*03:01 allele.

To confirm that the two class II AOA putatively causal variants do not contribute to class II COA risk, we repeated the analysis above for SNPs at the COA class II GWAS locus (Fig. 4B). When conditioning on either the AOA CS1 SNP rs9272346, the AOA CS2 HLA-DQA1*03:01 allele, or both, the COA associations remain genome-wide significant, although the magnitudes of the associations are reduced, likely due to including additional covariates in the model and the LD in the region (Additional file 1: Table S16). These results further support the argument that risk for COA and AOA are due to different causal variants in the HLA class II region.

Finally, we performed conditional analyses to assess the independence between the class I and class II signals. For each of the COA class I variants, we tested for their association with COA after conditioning on the tag class II SNPs and vice versa for the class II region. We similarly did this for AOA. For all results, the odds ratios (ORs) are largely similar and the 95% confidence intervals (CIs) overlap between the marginal and conditional associations, suggesting that the class I and class II signals are indeed independent (Additional file 1: Table S17).

### Replication of fine-mapping results

To replicate the COA and AOA putatively causal SNPs identified in the UK Biobank White British ancestry individuals, we examined a replication cohort of UK Biobank multi-ethnic individuals who were initially excluded from our studies. Asthma and allergy phenotypes were defined using the same criteria as in the discovery sample. The prevalence of both were similar in the discovery and replication samples (Additional file 1: Table S18, S19). To allow for allele frequency and effect size heterogeneity between the replication cohorts (*n*=43,449 White British, *n*=10,327 Asian or Asian British, *n*=7637 Black or Black British), we tested each variant for association with COA and AOA within each cohort and then performed a meta-analysis of the results. We required that the same allele is associated with asthma with the same direction of effect as in the discovery cohort. All of the variants, except the class II COA CS2 and the class I AOA CS1 SNPs, replicated at a significance threshold adjusted for multiple testing of 5.0 × 10^−3^ (Table 2, Additional file 1: Fig. S7, Additional file 2: Table S20). Additionally, the HLA-DQA1*03:01 allele had the most significant association for AOA compared to the other HLA alleles tested.

**Table 2:** Results of replication meta-analysis

Group	CS	Variant	Allele (non-risk/risk)	***p***-value	OR	95% CI
COA	Class I CS1	rs2428494	T/A	9.72 × 10^−04^	1.13	1.05–1.21
	Class I CS2	HLA-C p.11	Ser/Ala	2.66 × 10^−03^	1.16	1.05–1.29
	Class II CS1	rs28407950	T/C	1.30 × 10^−09^	1.29	1.19–1.40
	Class II CS2	rs35571244	T/C	2.74 × 10^−01^	1.08	0.94–1.25
AOA	Class I CS1	rs2428494	T/A	1.27 × 10^−02^	1.06	1.01–1.12
	Class II CS1	rs9274660	A/G	6.10 × 10^−08^	1.14	1.09–1.20
		rs3828789	G/T	4.59 × 10^−08^	1.15	1.09–1.20
		rs1063355	T/G	4.31 × 10^−08^	1.15	1.09–1.20
		rs9272346	G/A	7.52 × 10^−08^	1.14	1.09–1.20
	Class II CS2	*HLA-DQA1*	*03:01	1.27 × 10^−04^	1.13	1.06–1.21

*P*-values, odds ratios (ORs), and 95% confidence intervals (95% CI) in the replication cohorts are reported for each of the candidate variants from the discovery COA and AOA credible sets

Overall, all but two of the candidate variants (AOA rs2428494 and COA rs35571244) from the discovery cohort were significantly associated with COA or AOA in the replication cohort with the same direction of effect. Both variants were nominally associated with COA or AOA, with the same directions of effect, but were not significant after multiple test correction.

## Discussion

The HLA region is associated with more diseases than any other region of the genome [50], and variation in this region has been consistently associated with asthma risk in GWASs [2–5, 51–53]. However, the causal variants and genes have been unknown. Most previous large studies focused only on SNPs, which do not fully capture the extensive protein polymorphism at this locus. A recent study reported colocalizations between eQTLs for *HLA-B*, *HLA-DQB1*, *HLA-DQA1*, *HLA-DRA*, *TAP1*, and *RNF5* in induced pluripotent stem cells with asthma GWAS SNPs [45]. However, they did not separate COA and AOA, examine associations with HLA alleles or amino acids, or study gene expression in asthma-relevant cell types. Our study addressed these limitations and used relevant cell types and eQTL fine-mapping to identify putatively causal eQTLs. These studies extended our earlier observations of COA and AOA having both shared and distinct genetic risk to the HLA region. We also prioritized putatively causal variation by examining the location of associated amino acid variants within the functional domains of HLA proteins and of associated SNPs to functional annotations of gene regulation. In the class I region, we identified both a COA-specific association that may be mediated by HLA-C protein coding variation and a shared causal variant with unknown function. We did not find any shared causal variation between COA and AOA in the class II region, which was supported by replication studies. Nevertheless, our data strongly suggest that HLA class II-associated risk for AOA is mediated by both protein coding variation associated with the HLA-DQA1*03 alleles and differential expression of the nonclassical *HLA-DQA2* and *HLA-DQB2* genes.

Our fine-mapping studies in White British individuals from the UK Biobank revealed a lead GWAS SNP in an intron of *HLA-B* in the class I region that was putatively causal for both COA and AOA (rs2428494). However, rs2428494 was not in any eQTL credible sets. This SNP was also not an eQTL for any genes in GTEx tissues [54] or in immune cells in the Database of Immune Cell Expression, eQTLs, and Epigenomics (DICE) [55] and did not reside in ENCODE [48] *cis* regulatory elements (Additional file 1: Fig. S6). However, rs2428494 was predicted to be in flanking active TSSs for several T cell subsets in Roadmap [56]. Additionally, this association was replicated in a multi-ethnic cohort and was the lead class I region SNP in a meta-analysis of GWASs for allergic rhinitis, a common co-morbidity with asthma, with the same allele associated with risk [17]. Otherwise, little is known about this variant.

A second credible set in the class I region included a SNP (rs28481932) and an HLA-C amino acid polymorphism at position 11 and was specific to COA. We did not accrue any functional evidence for the SNP to mediate its effects through gene expression based on our eQTL studies and colocalizations with ENCODE annotations (Additional file 1: Fig. S6). However, the HLA-C amino acid polymorphism had a higher PIP and its location in the HLA-C protein makes it a promising functional candidate. This amino acid polymorphism was also associated with COA in the replication cohort. Along with other class I classical HLA genes, *HLA-C* is expressed on the cell surface of nearly all nucleated cells, where it presents intracellular peptides to the TCR of CD8 T cells. TCR recognition of foreign (e.g. viral) peptides presented by class I molecules activates these T cells, leading to the cytotoxic killing of infected cells by CD8 T cells and promoting inflammation. Position 11 lies in a β-sheet in the peptide-binding pocket, and the amino acid substitution may change the peptide-binding properties and alter antigen presentation and recognition by CD8 T cells. HLA-C is also a ligand for killer immunoglobulin receptors (KIRs) expressed on natural killer (NK) cells [57], which survey MHC class I levels on cell surfaces and can induce cell death when levels decrease during cell stress or viral infection [58]. Modulation of NK cell activity may be another potential role for this variant in COA [59, 60].

In the class II region, two credible sets were identified for COA, neither of which overlapped with the class II AOA credible sets. One of the credible sets (CS1, rs28407950) was replicated in the multi-ethnic cohort providing strong evidence that this SNP, or an untyped or rare variant in LD with it, is indeed a causal variant for COA. Additionally, we observed a nominally significant interaction between sex and rs28407950, with the risk variant having a stronger effect in females compared to males. Similarly, none of the class II COA CS2 SNPs were in eQTL credible sets and therefore not likely causal for resting gene expression in these cells. The fact that no HLA alleles or amino acids were included in either COA credible set largely rules out protein variation mediating risk at this locus for COA.

In contrast, fine-mapping studies identified the same causal variation underlying both AOA risk and expression of *HLA-DQA2* and *HLA-DQB2* genes at the class II region; these associations with AOA were replicated by fine-mapping studies in the multi-population cohort. While previous asthma GWAS have implicated the classical and highly polymorphic *HLA-DQA1* and *HLA-DQB1* class II genes [51, 53, 61], our study revealed associations with *HLA-DQA2* and *HLA-DQB2* genes in risk for AOA. The *HLA-DQA2* and *HLA-DQB2* genes are highly conserved paralogues of the *HLA-DQA1* and *HLA-DQB1* genes [62, 63], respectively, but are virtually devoid of amino acid polymorphisms in the peptide-binding pocket [62]. Little is known about the functions of the HLA-DQα2/HLA-DQβ2 protein, although it has been shown to form heterodimers in Langerhans cells and can present antigens and activate T cells [64]. It is notable that SNPs in the AOA CS1 were associated with increased *HLA-DQB2* and *HLA-DQA2* expression in different cell types, pointing to their potentially broad effects in both immune and airway epithelial tissues. The finding that asthma-associated SNPs in the class II region were associated with increased *HLA-DQA2* and *HLA-DQB2* expression is consistent with our earlier results showing that predicted increased expression of *HLA-DQA2* and *HLA-DQB2* [65] was among the most significant gene-based associations with asthma risk [3]. Our current study confirms this prediction and further implicates increased expression of these highly conserved and poorly characterized genes as potential mediators of risk for AOA. Moreover, a recent GWAS of asthma hospitalizations in White British adults from the UK Biobank also reported that the HLA class II region was the most significant association with hospitalizations [66]. Some of the associated SNPs were also eQTLs for HLA genes, including *HLA-DQA2*. This raises the possibility that the AOA class II CS1 variants in our study may also be associated with asthma hospitalizations and severity due to their effects on *HLA-DQA2* expression.

The HLA-DQA1*03 alleles may also play an important role in AOA risk. The class II AOA CS2 contained the HLA-DQA1*03:01 allele and five amino acids that define the HLA-DQA1*03 alleles; some of these polymorphisms are in regions with potential impact on peptide presentation and TCR interactions (Fig. 3C). We suggest therefore that HLA-DQA1*03:01 may be the causal variant for AOA in CS2. T cell activation and proliferation is driven by both differential expression levels and protein coding variation in the HLA genes which may affect binding affinities to different peptides [67, 68]. The HLA-DQA1*03:01 allele association was replicated, and it was the strongest HLA allele association in the replication cohort. Taken together, our studies indicate that increased expression of the *HLA-DQA2* and *HLA-DQB2* genes and coding variation in the HLA-DQA1*03 protein mediate the risks conferred by variation for AOA at the most significant GWAS locus [3].

Despite our delineation of specific HLA class I and class II variants and genes associated with risk for COA and AOA, our study had limitations. First, the fine-mapping method we used (SuSiE) has not yet been extended for logistic models or previously used in the HLA region. However, the use of linear methods to binary data can be justified (see Supplementary Methods) [22, 69–71], and our simulations indicate that SuSiE can accurately detect causal signals for binary traits (e.g. case-control status) as well as for quantitative traits (e.g. gene expression) in the genetically complex HLA region, at least in large sample sizes. Second, we did not identify potential mechanisms for all putatively causal signals for COA and AOA. In those cases, these variants may be eQTLs in other cell types not profiled in this study [72], in response to specific stimuli [73], or at specific developmental stages [74]. Further experimental evidence is needed to elucidate the relationships between the putatively causal variants identified in this study and their impacts on gene function and/or expression, and ultimately on risk for asthma. Third, not all of the variants in the credible sets replicated, which may be due to smaller sample size or may reflect true ethnic differences in risk alleles. The extensive diversity of HLA genes and haplotypes between worldwide populations and the known effects of local selection pressures on these genes makes multiancestry replication particularly challenging [75, 76], and large cohorts are needed to replicate the fine-mapping results. However, the single-variant association tests replicated nearly all representative variants in COA and AOA for class I and class II credible sets and the direction of effect was the same across discovery and replication cohorts. Given the importance of the HLA locus in asthma, additional studies need to be performed in larger cohorts from other racial and ethnic groups to gain a full picture of the roles of HLA genes in asthma. Finally, we discovered that HLA-DQA1*03:01 and a set of amino acids that are co-inherited on the HLA-DQA1*03 alleles were putatively causal for AOA. However, because the allele frequencies of the other HLA-DQA1*03 alleles (*03:02 and *03:03) were too infrequent in this sample to examine individually, we cannot determine if the effect is due solely to the HLA-DQA1*03:01 allele or a general effect of all *03 alleles.

In addition to age of asthma onset, many other known epidemiological factors distinguish asthma with onset in childhood compared to onset after puberty, including sex ratios, the importance of respiratory viral infections, and comorbidities with allergic diseases or obesity, as examples [77]. Future studies can determine whether these well-established differences in COA and AOA may also be explained, at least in part, by differences in causal variation in the HLA region identified in this study.

## Conclusions

Overall, our study highlights roles for both expression and protein coding variation in asthma risk. We suggest a prominent role for HLA-C protein coding variation in COA and both gene expression levels and protein coding changes in the HLA-DQ genes in AOA. We further propose that the HLA-DQA1*03:01 allele and SNPs that regulate the expression of the under-characterized *HLA-DQA2* and *HLA-DQB2* genes explain the class II HLA risk at the most significant AOA locus. Our study identified potential therapeutic targets for asthma and utilized a strategy that can serve as a model for fine-mapping other HLA-associated diseases by integrating approaches to narrow putatively causal variants and genes in the HLA region.

## Supplementary Information

**Additional file 1: Supplementary Methods. Fig. S1.** Ancestry PCs for the Replication and Discovery Cohorts. **Fig. S2.** HLA Allele Associations. **Fig. S3.** Amino Acid Associations. **Fig. S4.** Fine-Mapping Simulations in the HLA Region. **Fig. S5.** Expression of *HLA-DQB2* and *HLA-DQA2.* **Fig. S6.** ENCODE ChromHMM Results for SNPs in the Childhood-Onset and Adult-Onset Credible Sets. **Fig. S7.** Replication Results. **Table S1.** RNA-seq Sample Composition. **Table S6.** Putatively Causal Variants and Allergy. **Table S7.** Putatively Causal Variants and Sex. **Table S12.** HLA Region eQTLs. **Table S14.** Amino Acid Associations with the Highest PIPs in Each Credible Set. **Table S16.** Average r^2^ Between Childhood-Onset and Adult-Onset Asthma SNPs. **Table S17.** Marginal vs. Conditional Association Results. **Table S18.** Sample Composition of the Replication Cohort. **Table S19.** Self-Reported Ethnic Composition of the Replication Cohort.

**Additional file 2: Table S2.** HLA Allele Associations. **Table S3.** Allele Associations: Additive vs. Dominant Model. **Table S4.** HLA Heterogeneity Test. **Table S5.** HLA Amino Acid Polymorphism Associations. **Table S8.** SuSiE Credible Set Results. **Table S9.** HLA Allele Frequencies by Study. **Table S10.** List of SNPs in the Credible Sets Excluded from eQTL Analyses. **Table S11.** eQTL Results for All Credible Set SNPs. **Table S13.** eQTL Fine-Mapping Results. **Table S15.** Amino Acids in the Credible Sets and Their Corresponding HLA Alleles. **Table S20.** Replication Meta-Analysis Results.

## Data Availability

The study uses genotype and phenotype data from the UK Biobank under application number 44300. Access to the UK Biobank resource is available with application at http://www.ukbiobank.ac.uk and the summary statistics of COA and AOA by Pividori et al. can be downloaded from https://zenodo.org/record/3248979#.YTTfap5KjUJ [3, 78]. The gene expression datasets supporting this article are available on dbGaP https://www.ncbi.nlm.nih.gov/projects/gap/cgi-bin/study.cgi?study_id=phs000185.v7.p1 (Hutterite LCLs) and on the Gene Expression Omnibus (GEO) repository under the accession number GSE145505 (https://www.ncbi.nlm.nih.gov/geo/query/acc.cgi?acc=GSE145505) (URECA epithelial cells) and GSE96783 (https://www.ncbi.nlm.nih.gov/geo/query/acc.cgi?acc=GSE96783 ) (URECA PBMCs) [23–25].

## Abbreviations

GWAS: Genome-wide association study
HLA: Human leukocyte antigen
COA: Childhood-onset asthma
AOA: Adult-onset asthma
LD: Linkage disequilibrium
eQTL: Expression quantitative trait locus
MAF: Minor allele frequency
PC: Principal component
CI: Confidence interval
SuSiE: Sum of Single Effects
LCL: Lymphoblastoid cell line
PBMC: Peripheral blood mononuclear cell
NEC: Nasal epithelial cell
CS1: Credible Set 1
CS2: Credible Set 2
PIP: Posterior inclusion probability
TSS: Transcription start site
FDR: False discovery rate
TCR: T cell receptor
SD: Standard deviation
OR: Odds ratio
RAF: Risk allele frequency

## References

[1] Soriano JB, Kendrick PJ, Paulson KR, Gupta V, Abrams EM, Adedoyin RA (2020). Prevalence and attributable health burden of chronic respiratory diseases, 1990–2017: a systematic analysis for the Global Burden of Disease Study 2017. Lancet Respir Med.

[2] Demenais F, Margaritte-Jeannin P, Barnes KC, Cookson WOC, Altmüller J, Ang W (2018). Multiancestry association study identifies new asthma risk loci that colocalize with immune-cell enhancer marks. Nat Genet.

[3] Pividori M, Schoettler N, Nicolae DL, Ober C, Im HK (2019). Shared and distinct genetic risk factors for childhood-onset and adult-onset asthma: genome-wide and transcriptome-wide studies. Lancet Respir Med.

[4] Olafsdottir TA, Theodors F, Bjarnadottir K, Bjornsdottir US, Agustsdottir AB, Stefansson OA (2020). Eighty-eight variants highlight the role of T cell regulation and airway remodeling in asthma pathogenesis. Nat Commun.

[5] Ferreira MAR, Mathur R, Vonk JM, Szwajda A, Brumpton B, Granell R (2019). Genetic architectures of childhood- and adult-onset asthma are partly distinct. Am J Hum Genet.

[6] Daya M, Rafaels N, Brunetti TM, Chavan S, Levin AM, Shetty A (2019). Association study in African-admixed populations across the Americas recapitulates asthma risk loci in non-African populations. Nat Commun.

[7] Bycroft C, Freeman C, Petkova D, Band G, Elliott LT, Sharp K (2018). The UK Biobank resource with deep phenotyping and genomic data. Nature.

[8] Schoettler N, Rodríguez E, Weidinger S, Ober C (2019). Advances in asthma and allergic disease genetics: Is bigger always better?. J Allergy Clin Immunol.

[9] Simmonds M, Gough S (2009). The HLA region and autoimmune disease: associations and mechanisms of action. Curr Genomics.

[10] Mosaad YM (2015). Clinical role of human leukocyte antigen in health and disease. Scand J Immunol.

[11] Blackwell JM, Jamieson SE, Burgner D (2009). HLA and infectious diseases. Clin Microbiol Rev.

[12] Trowsdale J, Knight JC (2013). Major histocompatibility complex genomics and human disease. Annu Rev Genomics Hum Genet.

[13] Dendrou CA, Petersen J, Rossjohn J, Fugger L (2018). HLA variation and disease. Nat Rev Immunol.

[14] Band G, Marchini J. BGEN: A binary file format for imputed genotype and haplotype data. bioRxiv. 2018:308296. 10.1101/308296 [cited 19 Mar 2021].

[15] Motyer A, Vukcevic D, Cortes A, McVean G, Leslie S. Imputation of classical HLA types from UK Biobank genotype data. 2016.

[16] Jia X, Han B, Onengut-Gumuscu S, Chen W-M, Concannon PJ, Rich SS (2013). Imputing amino acid polymorphisms in human leukocyte antigens. PLoS One.

[17] Waage J, Standl M, Curtin JA, Jessen LE, Thorsen J, Tian C (2018). Genome-wide association and HLA fine-mapping studies identify risk loci and genetic pathways underlying allergic rhinitis. Nat Genet.

[18] Tian C, Hromatka BS, Kiefer AK, Eriksson N, Noble SM, Tung JY, et al. Genome-wide association and HLA region fine- mapping studies identify susceptibility loci for multiple common infections. Nat Commun. 2017;8 Available from: https://www.ncbi.nlm.nih.gov/pmc/articles/PMC5605711/pdf/41467_2017_Article_257.pdf [cited 15 Oct 2017]. 10.1038/s41467-017-00257-5 PMC5605711 28928442

[19] Church DM, Schneider VA, Graves T, Auger K, Cunningham F, Bouk N, et al. Modernizing reference genome assemblies. PLoS Biol. 2011;9(7) Available from: https://pubmed.ncbi.nlm.nih.gov/21750661/ [cited 25 Apr 2022]. 10.1371/journal.pbio.1001091 PMC3130012 21750661

[20] Genome Reference Consortium. Available from: https://www.ncbi.nlm.nih.gov/grc. [cited 26 Apr 2022].

[21] Willer CJ, Li Y, Abecasis GR (2010). METAL: fast and efficient meta-analysis of genomewide association scans. Bioinformatics.

[22] Wang G, Sarkar A, Carbonetto P, Stephens M (2020). A simple new approach to variable selection in regression, with application to genetic fine mapping. J R Stat Soc Ser B Stat Methodol.

[23] Cusanovich DA, Caliskan M, Billstrand C, Michelini K, Chavarria C, De Leon S (2016). Integrated analyses of gene expression and genetic association studies in a founder population. Hum Mol Genet.

[24] Altman MC, Whalen E, Togias A, O’Connor GT, Bacharier LB, Bloomberg GR (2018). Allergen-induced activation of natural killer cells represents an early-life immune response in the development of allergic asthma. J Allergy Clin Immunol.

[25] Altman MC, Calatroni A, Ramratnam S, Jackson DJ, Presnell S, Rosasco MG, et al. Endotype of allergic asthma with airway obstruction in urban children. J Allergy Clin Immunol. 2021; Available from: https://pubmed.ncbi.nlm.nih.gov/33713771/ [cited 3 Sep 2021]. 10.1016/j.jaci.2021.02.040 PMC8429519 33713771

[26] Gern JE, Visness CM, Gergen PJ, Wood RA, Bloomberg GR, O’Connor GT (2009). The Urban Environment and Childhood Asthma (URECA) birth cohort study: design, methods, and study population. BMC Pulm Med.

[27] Robinson J, Halliwell JA, Hayhurst JD, Flicek P, Parham P, Marsh SGE (2015). The IPD and IMGT/HLA database: allele variant databases. Nucleic Acids Res.

[28] Aguiar VRC, César J, Delaneau O, Dermitzakis ET, Meyer D (2019). Expression estimation and eQTL mapping for HLA genes with a personalized pipeline. PLoS Genet.

[29] Delaneau O, Ongen H, Brown AA, Fort A, Panousis NI, Dermitzakis ET (2017). A complete tool set for molecular QTL discovery and analysis. Nat Commun.

[30] Zhou X, Stephens M (2012). Genome-wide efficient mixed-model analysis for association studies. Nat Genet.

[31] Benjamini Y, Hochberg Y (1995). Controlling the false discovery rate: a practical and powerful approach to multiple testing. J R Stat Soc.

[32] Berman HM, Westbrook J, Feng Z, Gilliland G, Bhat TN, Weissig H (2000). The Protein Data Bank. Nucleic Acids Res.

[33] Kaur G, Gras S, Mobbs JI, Vivian JP, Cortes A, Barber T (2017). Structural and regulatory diversity shape HLA-C protein expression levels. Nat Commun.

[34] Jiang W, Birtley JR, Hung SC, Wang W, Chiou SH, Macaubas C (2019). In vivo clonal expansion and phenotypes of hypocretin-specific CD4+ T cells in narcolepsy patients and controls. Nat Commun.

[35] Tollefsen S, Hotta K, Chen X, Simonsen B, Swaminathan K, Mathews II (2012). Structural and functional studies of trans-encoded HLA-DQ2.3 (DQA1*03:01/DQB1*02:01) protein molecule. J Biol Chem.

[36] Schrödinger L, DeLano W. PyMOL [Internet]. 2020. Available from: http://www.pymol.org/pymol.

[37] HLA Nomenclature. 2019. Available from: http://hla.alleles.org/nomenclature/naming.html [cited 22 Feb 2021].

[38] Benner C, Havulinna AS, Järvelin MR, Salomaa V, Ripatti S, Pirinen M (2017). Prospects of fine-mapping trait-associated genomic regions by using summary statistics from genome-wide association studies. Am J Hum Genet.

[39] Westra H-J, Martínez-Bonet M, Onengut-Gumuscu S, Lee A, Luo Y, Teslovich N (2018). Fine-mapping and functional studies highlight potential causal variants for rheumatoid arthritis and type 1 diabetes. Nat Genet.

[40] Mahajan A, Taliun D, Thurner M, Robertson NR, Torres JM, Rayner NW (2018). Fine-mapping type 2 diabetes loci to single-variant resolution using high-density imputation and islet-specific epigenome maps. Nat Genet.

[41] Cruz-Tapias P, Castiblanco J, Anaya J-M, Levy RA, Anaya J-M, Shoenfeld Y, Rojas-Villarraga A, Cervera R (2013). HLA Association with autoimmune diseases. Autoimmunity: From Bench to Bedside.

[42] Jin Y, Roberts GHL, Ferrara TM, Ben S, Van Geel N, Wolkerstorfer A, et al. Early-onset autoimmune vitiligo associated with an enhancer variant haplotype that upregulates class II HLA expression. Nat Commun. 2019;10(1):–391. 10.1038/s41467-019-08337-4 [cited 31 Mar 2019]. 10.1038/s41467-019-08337-4 PMC6344500 30674883

[43] Raj P, Rai E, Song R, Khan S, Wakeland BE, Viswanathan K (2016). Regulatory polymorphisms modulate the expression of HLA class II molecules and promote autoimmunity. Elife..

[44] Apps R, Qi Y, Carlson JM, Chen H, Gao X, Thomas R (2013). Influence of HLA-C expression level on HIV control. Science (80- ).

[45] D’Antonio M, Reyna J, Jakubosky D, Donovan MKR, Bonder MJ, Matsui H (2019). Systematic genetic analysis of the MHC region reveals mechanistic underpinnings of HLA type associations with disease. Elife..

[46] Gutierrez-Arcelus M, Baglaenko Y, Arora J, Hannes S, Luo Y, Amariuta T (2020). Allele-specific expression changes dynamically during T cell activation in HLA and other autoimmune loci. Nat Genet.

[47] Kent WJ, Sugnet CW, Furey TS, Roskin KM, Pringle TH, Zahler AM (2002). The human genome browser at UCSC. Genome Res.

[48] Dunham I, Kundaje A, Aldred SF, Collins PJ, Davis CA, Doyle F (2012). An integrated encyclopedia of DNA elements in the human genome. Nature.

[49] Petersen J, Kooy-Winkelaar Y, Loh KL, Tran M, van Bergen J, Koning F (2016). Diverse T cell receptor gene usage in HLA-DQ8-associated celiac disease converges into a consensus binding solution. Structure..

[50] MacArthur J, Bowler E, Cerezo M, Gil L, Hall P, Hastings E (2017). The new NHGRI-EBI Catalog of published genome-wide association studies (GWAS Catalog). Nucleic Acids Res.

[51] Moffatt MF, Gut IG, Demenais F, Strachan DP, Bouzigon E, Heath S (2010). A large-scale, consortium-based genomewide association study of asthma. N Engl J Med.

[52] Li X, Howard TD, Zheng SL, Haselkorn T, Peters SP, Meyers DA (2010). Genome-wide association study of asthma identifies RAD50-IL13 and HLA-DR/DQ regions. J Allergy Clin Immunol.

[53] Lasky-Su J, Himes BE, Raby BA, Klanderman BJ, Sylvia JS, Lange C (2012). HLA-DQ strikes again: genome-wide association study further confirms HLA-DQ in the diagnosis of asthma among adults. Clin Exp Allergy.

[54] Aguet F, Brown AA, Castel SE, Davis JR, He Y, Jo B (2017). Genetic effects on gene expression across human tissues. Nature..

[55] Schmiedel BJ, Singh D, Madrigal A, Valdovino-Gonzalez AG, White BM, Zapardiel-Gonzalo J (2018). Impact of genetic polymorphisms on human immune cell gene expression. Cell..

[56] Kundaje A, Meuleman W, Ernst J, Bilenky M, Yen A, Roadmap Epigenomics Consortium (2015). Integrative analysis of 111 reference human epigenomes. Nature..

[57] Blais ME, Dong T, Rowland-Jones S (2011). HLA-C as a mediator of natural killer and T-cell activation: spectator or key player?. Immunology.

[58] Fadda L, Borhis G, Ahmed P, Cheent K, Pageon SV, Cazaly A (2010). Peptide antagonism as a mechanism for NK cell activation. Proc Natl Acad Sci U S A.

[59] Karimi K, Forsythe P (2013). Natural killer cells in asthma. Front Immunol.

[60] Kim JH, Jang YJ (2018). Role of natural killer cells in airway inflammation. Allergy Asthma Immunol Res.

[61] Li X, Ampleford EJ, Howard TD, Moore WC, Torgerson DG, Li H (2012). Genome-wide association studies of asthma indicate opposite immunopathogenesis direction from autoimmune diseases. J Allergy Clin Immunol.

[62] Berdoz J, Tiercy J-M, Rollini P, Mach B, Gorski J (1989). Remarkable sequence conservation of the HLA-DQB2 locus (DX beta) within the highly polymorphicDQ subregion of the human MHC. Immunogenetics.

[63] Gaur LK, Heise ER, Thurtle PS, Nepom GT (1992). Conservation of the HLA-DQB2 locus in nonhuman primates. J Immunol.

[64] Lenormand C, Bausinger H, Gross F, Signorino-Gelo F, Koch S, Peressin M (2012). HLA-DQA2 and HLA-DQB2 genes are specifically expressed in human Langerhans cells and encode a new HLA class II molecule. J Immunol.

[65] Gamazon ER, Wheeler HE, Shah KP, Mozaffari SV, Aquino-Michaels K, Carroll RJ (2015). A gene-based association method for mapping traits using reference transcriptome data. Nat Genet.

[66] Yan Q, Forno E, Herrera-Luis E, Pino-Yanes M, Yang G, Oh S, et al. A genome-wide association study of asthma hospitalizations in adults. J Allergy Clin Immunol. 2021;147(3):933–40. 10.1016/j.jaci.2020.08.020 PMC7921212 32890573

[67] Mine KL, Tedesco-Silva H, Mourão TB, Campos EF, Salzedas LA, Aguiar B (2018). Heightened expression of HLA-DQB1 and HLA-DQB2 in pre-implantation biopsies predicts poor late kidney graft function. Hum Immunol.

[68] Farina F, Picascia S, Pisapia L, Barba P, Vitale S, Franzese A (2019). HLA-DQA1 and HLA-DQB1 alleles, conferring susceptibility to celiac disease and type 1 diabetes, are more expressed than non-predisposing alleles and are coordinately regulated. Cells.

[69] Pirinen M, Donnelly P, Spencer CCA (2013). Efficient computation with a linear mixed model on large-scale data sets with applications to genetic studies. Ann Appl Stat.

[70] Benner C, Spencer CCA, Havulinna AS, Salomaa V, Ripatti S, Pirinen M (2016). FINEMAP: efficient variable selection using summary data from genome-wide association studies. Bioinformatics.

[71] Banerjee S, Zeng L, Schunkert H, Söding J (2018). Bayesian multiple logistic regression for case-control GWAS. PLoS Genet.

[72] Aguet F, Barbeira AN, Bonazzola R, Brown A, Castel SE, Jo B (2020). The GTEx Consortium atlas of genetic regulatory effects across human tissues. Science.

[73] Calderon D, Nguyen MLT, Mezger A, Kathiria A, Müller F, Nguyen V (2019). Landscape of stimulation-responsive chromatin across diverse human immune cells. Nat Genet.

[74] Zhang S, Zhang H, Zhou Y, Qiao M, Zhao S, Kozlova A (2020). Allele-specific open chromatin in human iPSC neurons elucidates functional disease variants. Science (80- ).

[75] Gourraud P-A, Khankhanian P, Cereb N, Yang SY, Feolo M, Maiers M (2014). HLA diversity in the 1000 genomes dataset. PLoS One.

[76] Luo Y, Kanai M, Choi W, Li X, Yamamoto K, Ogawa K, et al. A high-resolution HLA reference panel capturing global population diversity enables multi-ethnic fine-mapping in HIV host response. medRxiv. 2020. 10.1101/2020.07.16.20155606 [cited 10 Dec 2020].

[77] Trivedi M, Denton E (2019). Asthma in children and adults—what are the differences and what can they tell us about asthma?. Front Pediatr.

[78] Pividori M, Schoettler N, Nicolae DL, Ober C, Im HK. Shared and distinct genetic risk factors for childhood-onset and adult-onset asthma: genome-wide and transcriptome-wide studies [Data set]: Zenodo; 2019. 10.5281/zenodo.3248979. 10.1016/S2213-2600(19)30055-4 PMC6534440 31036433

