# Supplementary material for: Fine-mapping studies distinguish genetic risks for childhood- and adult-onset asthma in the HLA region
Source: Genome Med. 2022 May 24;14:55. doi: 10.1186/s13073-022-01058-2 (PMC9128203; doi:10.1186/s13073-022-01058-2)
Supplement: Supplementary file 1 — Additional file 1: Supplementary Methods. Fig. S1. Ancestry PCs for the Replication and Discovery Cohorts. Fig. S2. HLA Allele Associations. Fig. S3. Amino Acid Associations. Fig. S4. Fine-Mapping Simulations in the HLA Region. Fig. S5. Expression of HLA-DQB2 and HLA-DQA2. Fig. S6. ENCODE ChromHMM Results for SNPs in the Childhood-Onset and Adult-Onset Credible Sets. Fig. S7. Replication Results. Table S1. RNA-seq Sample Composition. Table S6. Putatively Causal Variants and Allergy. Table S7. Putatively Causal Variants and Sex. Table S12. HLA Region eQTLs. Table S14. Amino Acid Associations with the Highest PIPs in Each Credible Set. Table S16. Average r2 Between Childhood-Onset and Adult-Onset Asthma SNPs. Table S17. Marginal vs. Conditional Association Results. Table S18. Sample Composition of the Replication Cohort. Table S19. Self-Reported Ethnic Composition of the Replication Cohort. [file 13073_2022_1058_MOESM1_ESM.pdf]

**Fine-Mapping Studies Distinguish Genetic Risks for Childhood- and Adult-Onset Asthma in the HLA Region**

**Supplementary Methods, Figures, and Tables**

Selene M Clay[1*], Nathan Schoettler[2], Andrew M Goldstein[3], Peter Carbonetto[1], Matthew Dapas[1], Matthew C Altman[4,5], Mario G Rosasco[5], James E Gern[6], Daniel J Jackson[6], Hae Kyung Im[7], Matthew Stephens[3], Dan L Nicolae[1,3], Carole Ober[1*]

[1]Department of Human Genetics, University of Chicago, Chicago, IL 60637 USA

[2]Section of Pulmonary and Critical Care, Department of Medicine, University of Chicago, Chicago, IL 60637 USA

[3]Department of Statistics, University of Chicago, Chicago, IL 60637 USA

[4]Division of Allergy and Infectious Diseases, Department of Medicine, University of Washington, Seattle, WA, USA

[5]Systems Immunology Program, Benaroya Research Institute, Seattle, WA, USA

[6]Department of Pediatrics, University of Wisconsin, School of Medicine and Public Health, Madison, WI 53706 USA

[7]Section of Genetic Medicine, Department of Medicine, University of Chicago, Chicago, IL 60637 USA

## Table of Contents

**(3) Supplementary Methods**

- (4) Fine Mapping the HLA Region
- (4) HLA Fine-Mapping Simulations
- (6) Gene Expression and eQTL Studies

**(8) Supplementary Figures**

- (9) Fig. S1. Ancestry PCs for the Replication and Discovery Cohorts
- (10) Fig. S2. HLA Allele Associations
- (11) Fig. S3. Amino Acid Associations
- (13) Fig. S4. Fine-Mapping Simulations in the HLA Region
- (15) Fig. S5. Expression of *HLA-DQB2* and *HLA-DQA2*
- (16) Fig. S6. ENCODE ChromHMM Results for SNPs in the Childhood-Onset and Adult-Onset Credible Sets
- (18) Fig. S7. Replication Results

**(20) Supplementary Tables**

- (21) Table S1. RNA-seq Sample Composition
- (22) Table S2. HLA Allele Associations*
- (23) Table S3. Allele Associations: Additive vs. Dominant Model*
- (24) Table S4. HLA Heterogeneity Test*
- (25) Table S5. HLA Amino Acid Polymorphism Associations*
- (26) Table S6. Putatively Causal Variants and Allergy
- (27) Table S7. Putatively Causal Variants and Sex
- (28) Table S8. SuSiE Credible Set Results*
- (29) Table S9. HLA Allele Frequencies by Study*
- (30) Table S10. List of SNPs in the Credible Sets Excluded from eQTL Analyses*
- (31) Table S11. eQTL Results for All Credible Set SNPs*
- (32) Table S12. HLA Region eQTLs
- (33) Table S13. eQTL Fine-Mapping Results*
- (34) Table S14. Amino Acid Associations with the Highest PIPs in Each Credible Set
- (35) Table S15. Amino Acids in the Credible Sets and Their Corresponding HLA Alleles*
- (36) Table S16. Average $r^2$ Between Childhood-Onset and Adult-Onset Asthma SNPs
- (37) Table S17. Marginal vs. Conditional Association Results
- (38) Table S18. Sample Composition of the Replication Cohort
- (39) Table S19. Self-Reported Ethnic Composition of the Replication Cohort
- (40) Table S20. Replication Meta-Analysis Results*

* Tables are available in Additional File 2

**(41) Supplementary References**

## Supplementary Methods

## Fine Mapping the HLA Region

We used Sum of Single Effects (SuSiE(1)) (susieR R package version 0.9.0) to fine map the HLA loci for childhood-onset asthma (COA) and adult-onset asthma (AOA). The susieR R package does not currently allow for the inclusion of covariates, so sex and the first 10 ancestral principal components (PCs) were regressed out of the genotype matrix and phenotype vector using linear regression; we used the residuals of the genotype matrix and phenotype vector as inputs to SuSIE. The SuSiE method is based on a linear regression, and so when applied to binary data, it will estimate and test for effects in terms of risk differences, rather than the more conventional odds ratio (OR). Applying linear methods to binary data is justified here because the estimated ORs were all small (<1.3), the allele frequencies were not too extreme, and the sample size (here, limited by the smaller number of cases) was large (2–4). See Pirinen *et al.* Section 3 for detailed discussion of applying linear methods to binary data and the relationship between estimated risk differences and ORs. We assumed at most $L$ = 10 causal variants and set susieR to estimate the residual and prior variances. We retained only level-95% credible sets (coverage = 0.95). We took the additional step of discarding credible sets in which the "purity" (smallest absolute correlation among all pairs of variants within the credible set) was less than 0.50. We only considered credible sets that contained at least one variant reaching genome-wide significance to avoid any possible artifacts.

## HLA Fine-Mapping Simulations

Because the HLA region is extraordinarily complex, we assessed the performance of SuSiE in this region by simulation. Existing genotype and covariate data were used to leverage the true correlation structure in the class I and class II regions to simulate both binary (e.g. case/control status) and quantitative (e.g. gene expression) outcomes.

To simulate binary (e.g. case/control status) outcomes, we used the genotype matrix *X* (HLA class I or class II loci defined by Pividori *et al.*(5)) and covariate matrix *Z* from the UK Biobank and set the individual-level log-odds of asthma to be

$$\ln \frac{p_i}{1-p_i} = \sum_j \beta_j X_{ij} + \sum_k \delta_k Z_{ik} + \alpha$$

for individual *i*, SNPs *j*, covariates *k*, fixed effect vectors $\beta$ and $\delta$, and a fixed intercept $\alpha$. We used the true matrix of covariates and covariate effects $\delta$ estimated from a logistic regression, separately for COA and AOA simulations. $\beta_j$ was set to 0 for all non-causal variants. For causal variants, $\beta_j$was set using effect sizes similar to what was found in the Pividori COA and AOA GWASs(5). We randomly selected 0-3 variants from a random uniform distribution to be causal (with non-zero effects) for both the class I and class II regions using both COA and AOA effect sizes. We simulated case/control status for each individual as $Y_i \sim Bernoulli(p_i)$ independently and regressed out the covariates in Z from *X* and *Y*.

For quantitative outcomes (e.g. gene expression), we used *X* and *Z* in the HLA class I and HLA class II regions from the nasal epithelial cell (NEC) dataset from URECA described below. We set the individual-level mean to be

$$\mu_i = \sum_j \beta_j X_{ij} + \sum_k \delta_k Z_{ik} + \alpha$$

and we used the true matrix of covariates and effects $\delta$ estimated from a linear regression. $\beta_j$ was set to 0 for all non-causal variants, and causal $\beta_j$were set using effect sizes similar to what was found from the NEC eQTL studies (described below). We similarly randomly selected 0-3 causal signals in both the class I and class II regions and set the individual level response to be $Y_i \sim N(\mu_i, \sigma^2)$. We similarly regressed out the covariates in Z from X and Y. These simulations were used to test how well SuSiE recovers the causal effects over each simulation.

**Gene Expression and eQTL Studies**

*Lymphoblastoid Cell Lines (LCLs)*

We examined RNA-seq data previously collected from LCLs from 398 Hutterites(6). The Hutterites are a founder population of European descent with well characterized HLA types for the polymorphic *HLA-A*, *HLA-B*, *HLA-C*, *HLA-E*, *HLA-G*, *HLA-DPB1*, *HLA-DRB1*, *HLA-DQB1*, and *HLA-DQA1* genes(7). The sample was composed of 191 males and 207 females who were between the ages of 10 and 60 at the time of sample collection. Informed consent was obtained from all participants under University of Chicago IRB-approved protocols.

Standard RNA-seq pipelines that map reads to a reference genome can provide biased expression estimates at the highly polymorphic HLA loci due to the potentially large number of differences between the sequence of an individual's HLA type and the reference sequence used for mapping(8,9). Expression estimates can be improved by mapping RNA-seq reads to the sequences for each individual's known HLA type(8). For the polymorphic HLA genes, we aligned RNA-seq reads to reference sequences from the IMGT database(10) for each individual's known HLA type, removing duplicate reads with WASP(11). Sequencing reads were mapped and quantified using STAR/2.6.1(12) for other genes. Samples with >7M uniquely mapped reads underwent trimmed means of M-value (TMM) normalization and voom transformation(13). We corrected for extraction date and sequencing batch with limma(14).

To perform eQTL mapping, associations between SNPs and expression of genes in the HLA class I and class II regions were performed with Genome-wide Efficient Mixed Model Association (GEMMA)(15) using a kinship matrix to correct for relatedness between Hutterite individuals. We used a linear mixed model (LMM) including age and sex as covariates and considered all variants within 1 Mb of the transcription start site (TSS) of each expressed gene.

*Peripheral Blood Mononuclear Cells (PBMCs)*

We examined unstimulated PBMC RNA-seq data from 132 (78 males, 54 females) African-American children from the URban Environment and Childhood Asthma (URECA) birth

cohort who were 2 years old at the time of sample collection(16,17). Whole genome sequencing (WGS) was performed using the Illumina NovaSEQ6000 with 150 bp paired-end reads. Reads were aligned to the GRCh38 human reference genome (including alternate loci and decoy contigs) using BWA-MEM(18) (Burrows-Wheeler Aligner; v0.7.17). Aligned reads underwent duplicate removal (Picard MarkDuplicates v2.8.1) and base quality score recalibration (GATK BaseRecalibrator; v3.8) against known sites (dbSNP138, known indels, and Mills and 1 KG gold standard indels) provided in the GATK resource bundle(19). Reads that mapped to the primary HLA region (chr6:28510120-33480577), reads that mapped to the GRCh38 HLA contigs, and unmapped reads were used for WGS HLA typing. We used HLA-LA(20) to infer HLA types from WGS for *HLA-A, HLA-B, HLA-C, HLA-E, HLA-F, HLA-DRB1, HLA-DQB1, HLA-DQA1, HLA-DPB1,* and *HLA-DPA1*. Reads were mapped and normalized as previously described. To perform eQTL mapping, we examined linear regressions with QTLtools(21), using a nominal pass and *cis*-window size of 1 Mb around the TSS. We included sex, collection site, the first three ancestral PCs, and 19 latent factors(23) to account for unwanted variation as covariates in the analysis.

*Nasal Epithelial Cells (NECs)*

We examined NEC RNA-seq data from 188 (92 females, 96 males) African-American children (age 11 at time of sample collection) from the URECA cohort(24). As described above for the PBMCs, we used HLA-LA to infer HLA types from whole-genome sequences, mapped RNA-seq reads as described above, and used QTLtools to perform eQTL mapping, using sex, the first three ancestral PCs, collection site, epithelial cell proportion, sequencing batch, and seven latent factors(23) as covariates in the analysis.

**Supplementary Figures**

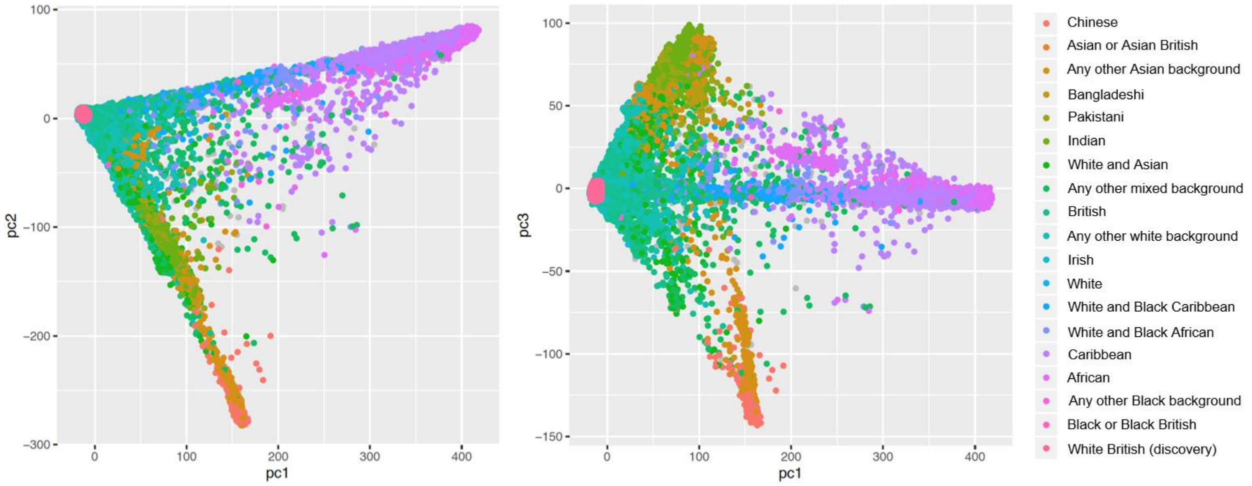


**Fig. S1. Ancestry PCs for the Replication and Discovery Cohorts.**

Ancestry PC1, PC2, and PC3 are shown for the discovery cohort ("White British (discovery)") and the replication cohort, with the colors corresponding to self-reported ancestry.

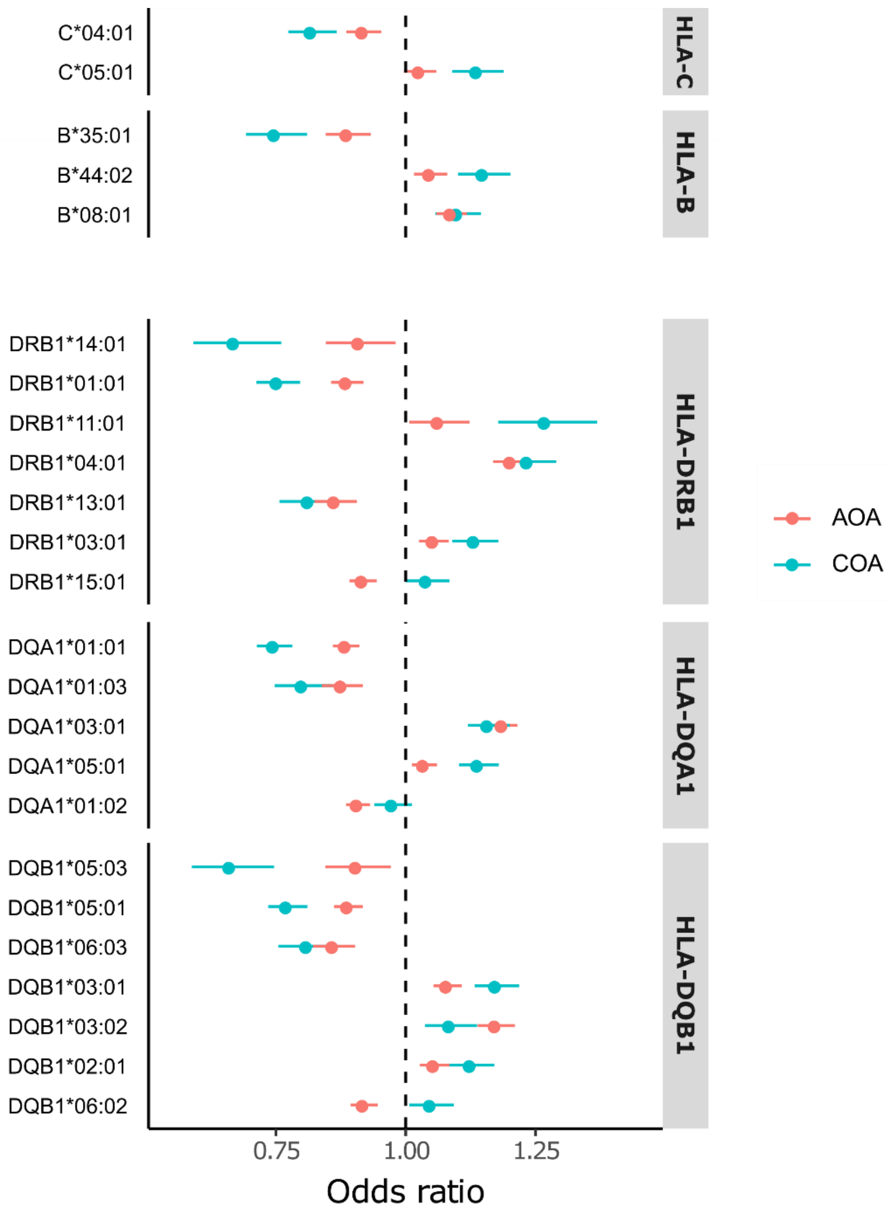


**Fig. S2. HLA Allele Associations.**

Odds ratios and 95% confidence intervals are shown for the HLA alleles that were significantly associated ($p<5.0x10^{-8}$) with either childhood-onset asthma (COA, blue) and/or adult-onset asthma (AOA, red). The results for all alleles for the six HLA loci are shown in Table S2.

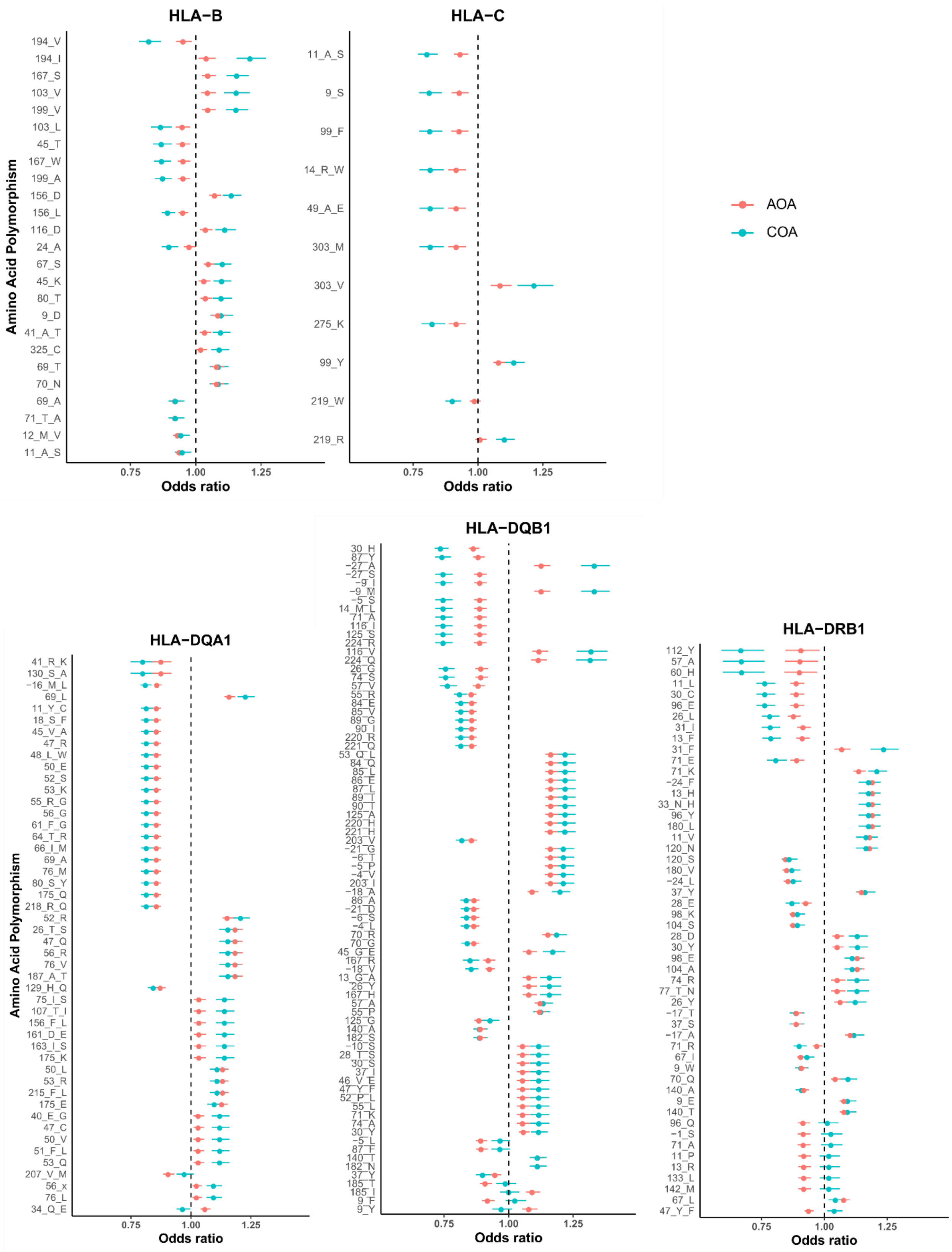


**Fig. S3. Amino Acid Associations.**

Odds ratios and 95% confidence intervals are shown for HLA amino acid polymorphisms that were significantly associated ($p<5x10^{-8}$) with either childhood-onset asthma (COA, blue) and/or adult-onset asthma (AOA, red).

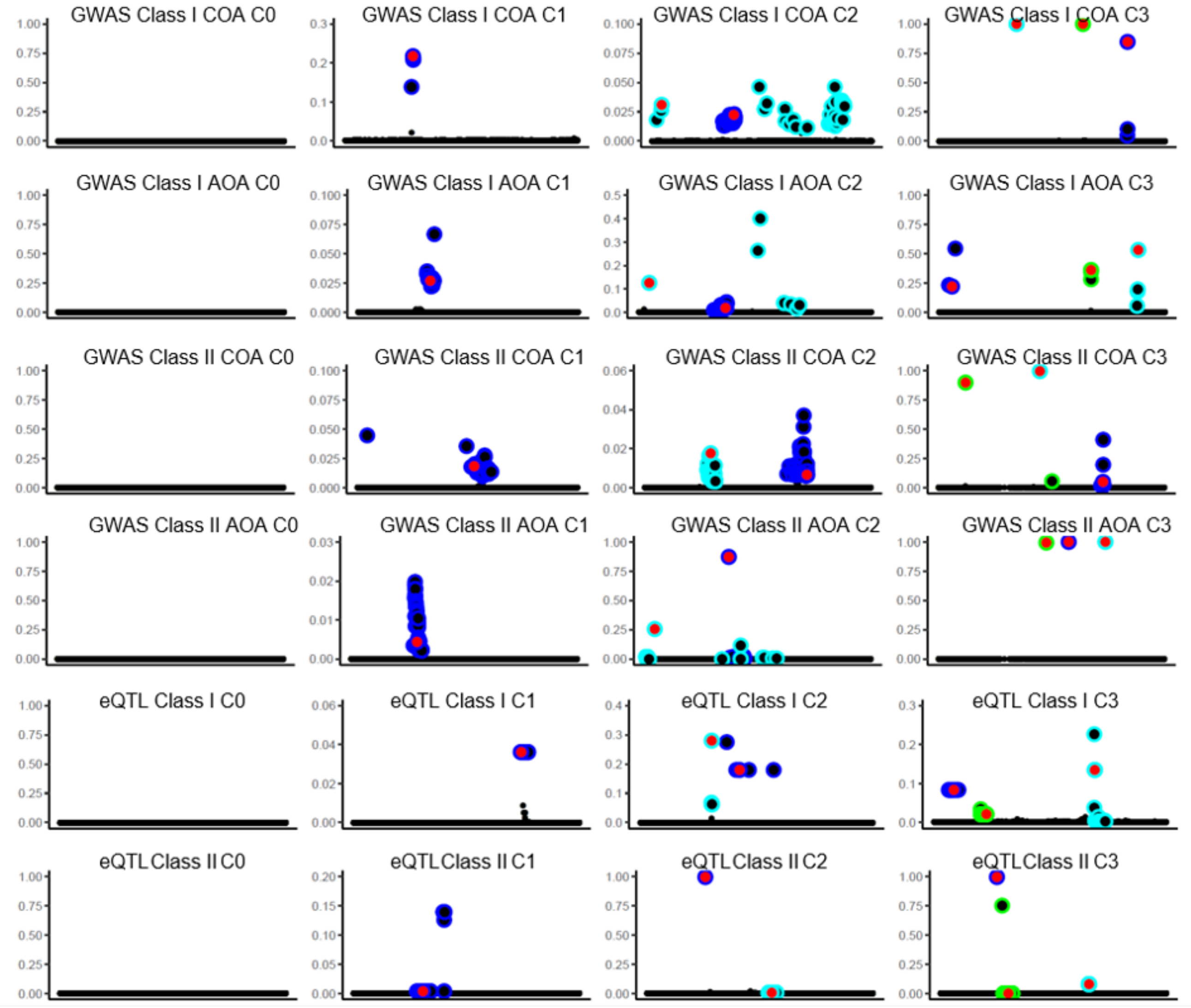


**Fig. S4. Fine-Mapping Simulations in the HLA Region.**

Each panel is a simulation. The top four rows are the simulated binary (“GWAS”) traits and the bottom two rows are the simulated quantitative (“eQTL”) traits. Simulations were performed for both the class I and class II regions separately. The binary outcomes were also simulated using covariate effects estimated for either childhood-onset asthma (COA) or adult-onset asthma (AOA) (see Supplementary Methods for more details). C refers to the number of causal variants (0-3). The colors represent the credible sets detected by SuSiE, with the designated causal

effect variant(s) in red. SuSiE correctly identified the accurate number of causal signals and reported a true causal signal in each credible set in all the simulations.

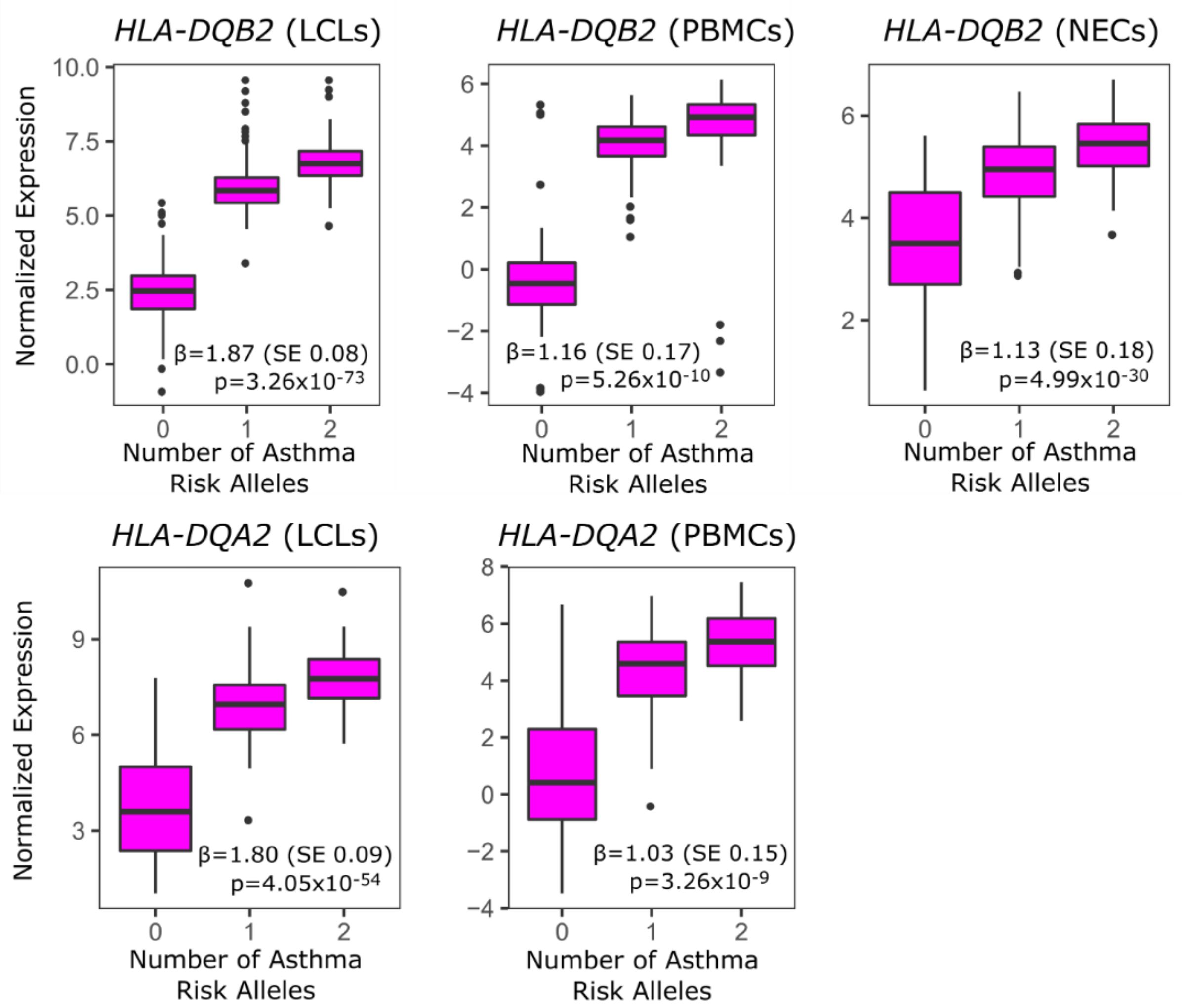


**Fig. S5. Expression of *HLA-DQB2* and *HLA-DQA2*.**

Normalized expression of each gene by the number of asthma-risk alleles for rs9272346 (A) for lymphoblastoid cell lines (LCLs) and peripheral blood mononuclear cells (PBMCs) and rs9274660 (G) for nasal epithelial cells (NECs), which were representative class II AOA CS1 SNPs.

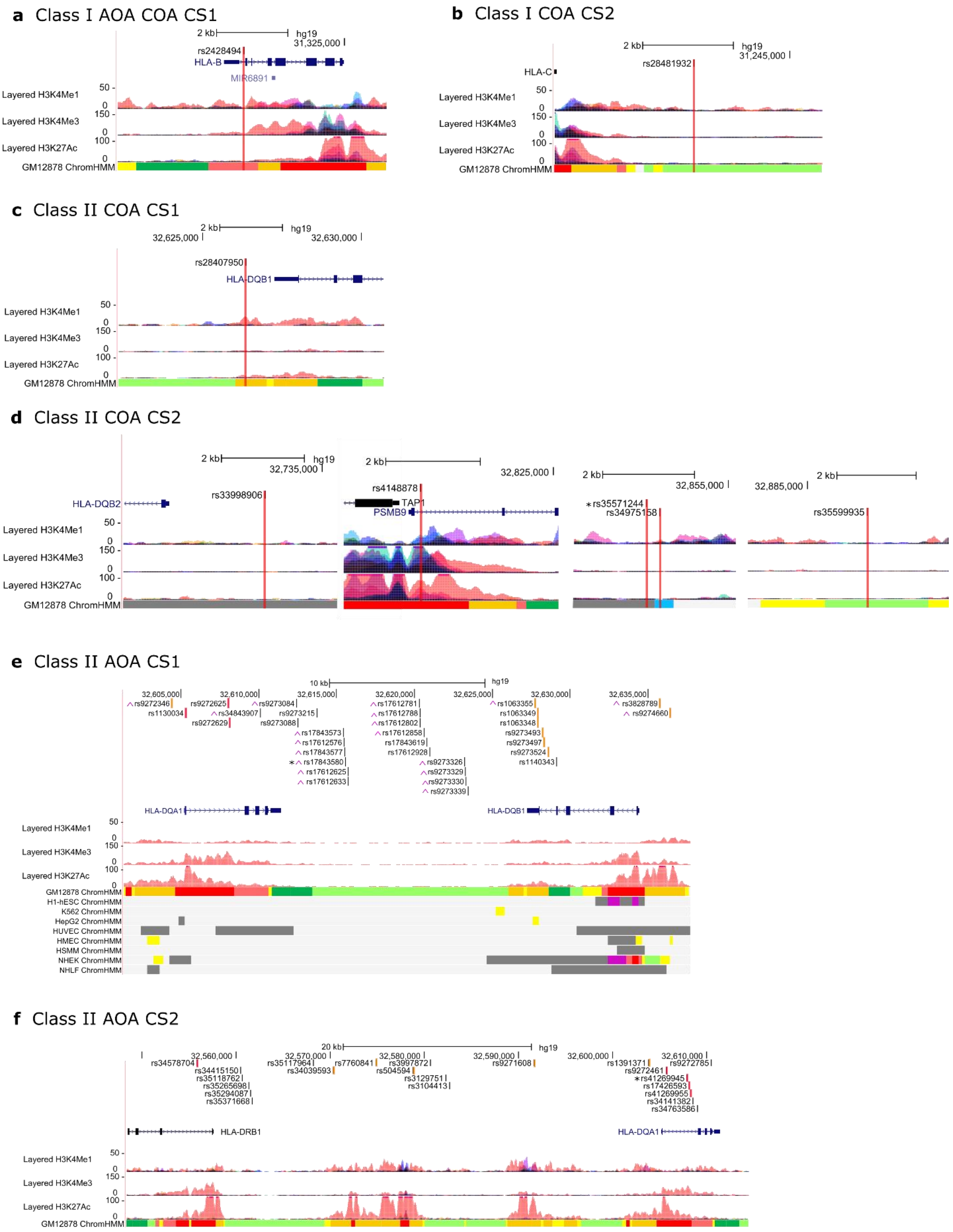


**Fig. S6. ENCODE ChromHMM Results for SNPs in the Childhood-Onset Asthma and**

**Adult-Onset Asthma Credible Sets**.

Vertical red line indicates the location of each SNP. Layered H3K4Me1, H3K4ME3, and H3K27Ac marks and ChromHMM states are shown for the GM12878 cells. Red: active promoter, light red: weak promoter, purple: inactive/poised promoter, orange: strong enhancer, yellow: weak/poised enhancer, blue: insulator, dark green: transcriptional transition/elongation, light green: weak transcribed, gray: polycomb-repressed, light gray: heterochromatin/low signal. Asterisk denotes rsid with the highest PIP. **a)** rs2428494 (shared class I CS) was predicted to reside in a weak promoter, **b)** rs28481932 (class I COA CS2) in a weakly transcribed region, and **c)** rs28407950 (class II COA CS1) in an strong enhancer. **d)** Class II COA CS2 SNPs were predicted to reside in polycomb-repressed, active promoter, polycomb-repressed, insulator, and weakly transcribed regions (from left to right). **e)** Class II AOA CS1 SNPs (see main manuscript for a discussion of these results). The red or orange mark next to the rsid indicates it is predicted to reside in an active promoter or strong enhancer, respectively. Magenta ^ indicates if it was an eQTL in our study. **f)** Class II AOA CS2 SNPs. Figures created from http://genome.uscs.edu(25).

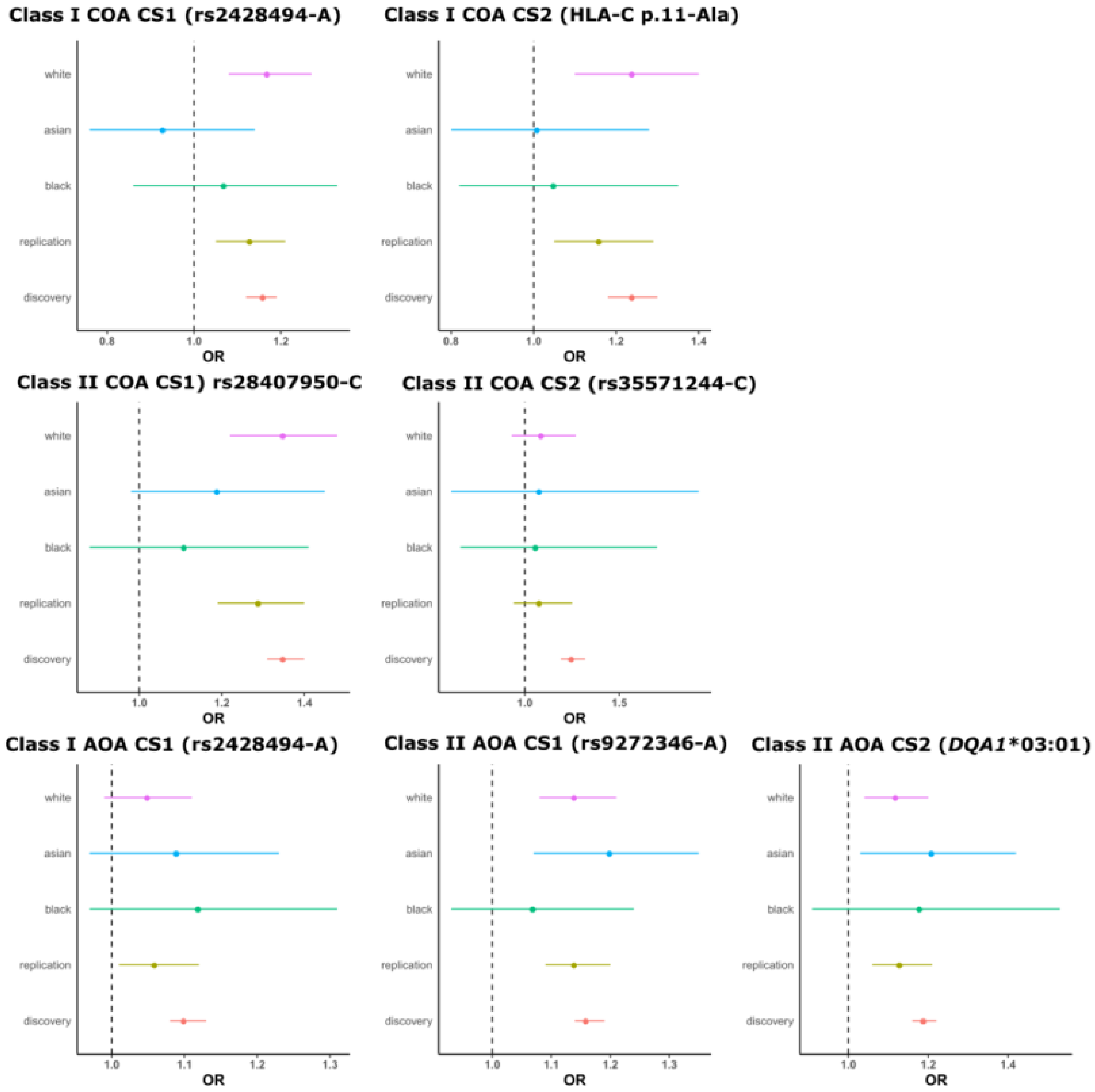


**Fig. S7. Replication Results.**

Odds ratios and 95% confidence intervals are shown for the candidate variant allele or amino acid polymorphism in the discovery COA and AOA CSs for self-reported White (British, Irish, White, Any other White background), self-reported Black or Black British, self-reported Asian or

Asian British, entire replication cohort (consisting of White, Black or Black British, Asian or Asian British), and the White British discovery cohort.

## Supplementary Tables

**Table S1. RNA-seq Sample Composition.**

| Cells | Study | Ancestry | Sample Size | Age (yrs) | Sex |
|---|---|---|---|---|---|
| LCLs (EBV-transformed B cells) | Hutterites | European American | 398 | 10-60 | 191 M, 207 F |
| Peripheral blood mononuclear cells (PBMCs) | URECA | African American | 132 | 2 | 78 M, 54 F |
| Nasal epithelial cells (NECs) | URECA | African American | 188 | 11 | 96 M, 92 F |

Sample composition from RNA-seq data collected from LCLs (Hutterites(6)) and the PBMCs(16,17) and NECs(24) from URECA (URban Environment and Childhood Asthma).

**Table S2. HLA Allele Associations**

See Additional File 2

**Table S3. Allele Associations: Additive vs. Dominant Model**

See Additional File 2

**Table S4. HLA Heterogeneity Test**

See Additional File 2

**Table S5. HLA Amino Acid Polymorphism Associations**

See Additional File 2

## Table S6. Putatively Causal Variants and Allergy

| CS | Var | Original p-value | Original OR | Allergy Cov p-value | Allergy Cov OR | No allergy p-value | No allergy OR | Interaction p-value |
|---|---|---|---|---|---|---|---|---|
| Childhood-Onset Asthma | | | | | | | | |
| Class I CS1 | rs2428494 | $8.77x10^{-23}$ | 1.157 | $4.97x10^{-21}$ | 1.151 | $6.11x10^{-16}$ | 1.160 | 0.494 |
| Class I CS2 | HLA-C p.11 | $3.12x10\text{-}^{19}$ | 1.241 | $2.12x10^{-18}$ | 1.236 | $7.84x10^{-12}$ | 1.224 | 0.562 |
| Class II CS1 | rs28407950 | $1.37x10\text{-}^{59}$ | 1.355 | $1.31x10^{-54}$ | 1.339 | $7.74x10^{-41}$ | 1.361 | 0.219 |
| Class II CS2 | rs35571244 | $1.07x10^{-17}$ | 1.253 | $3.21x10^{-16}$ | 1.242 | $2.78x10^{-11}$ | 1.243 | 0.9995 |
| Adult-Onset Asthma | | | | | | | | |
| Class I CS1 | rs2428494 | $4.52x10^{-23}$ | 1.104 | $3.13x10^{-21}$ | 1.100 | $1.66x10^{-16}$ | 1.099 | 0.939 |
| Class II CS1 | rs9272346 | $1.98x10^{-47}$ | 1.163 | $1.42x10^{-47}$ | 1.164 | $1.82x10^{-37}$ | 1.166 | 0.77 |
| Class II CS2 | DQA1*0301 | $6.91x10^{-47}$ | 1.187 | $1.72x10^{-48}$ | 1.202 | $1.62x10^{-40}$ | 1.211 | 0.352 |

Each row is a result for each variant reflecting each credible set (CS). The original p-value and odds ratio (OR) for the risk allele are shown, then the p-value and OR when allergy was included as a covariate in the regression ("Allergy Cov"), when all individuals with allergy were excluded ("No allergy"), and the interaction between the variant and allergy status are shown.

**Table S7. Putatively Causal Variants and Sex**

| CS | Var | Original p-value | Original OR | Females p-value | Females OR | Males p-value | Males OR | Interaction p-value |
|---|---|---|---|---|---|---|---|---|
| Childhood-Onset Asthma | | | | | | | | |
| Class I CS1 | rs2428494 | $8.77x10^{-23}$ | 1.157 | $3.46x10^{-09}$ | 1.146 | $3.71x10^{-15}$ | 1.164 | 0.613 |
| Class I CS2 | HLA-C p.11 | $3.12x10^{-19}$ | 1.241 | $4.59x10^{-12}$ | 1.302 | $3.68x10^{-09}$ | 1.200 | 0.098 |
| Class II CS1 | rs28407950 | $1.37x10^{-59}$ | 1.355 | $2.87x10^{-33}$ | 1.426 | $6.08x10^{-29}$ | 1.308 | 0.024 |
| Class II CS2 | rs35571244 | $1.07x10\text{-}^{17}$ | 1.253 | $3.85x10^{-08}$ | 1.253 | $4.94x10^{-11}$ | 1.253 | 0.996 |
| Adult-Onset Asthma | | | | | | | | |
| Class I CS1 | rs2428494 | $4.52x10^{-23}$ | 1.104 | $6.63x10^{-14}$ | 1.099 | $9.69x10^{-11}$ | 1.112 | 0.575 |
| Class II CS1 | rs9272346 | $1.98x10^{-47}$ | 1.163 | $3.02x10^{-31}$ | 1.165 | $7.18x10^{-18}$ | 1.159 | 0.812 |
| Class II CS2 | DQA1*0301 | $6.91x10^{-47}$ | 1.187 | $1.45x10^{-34}$ | 1.202 | $2.32x10^{-14}$ | 1.162 | 0.174 |

For each variant, the p-value and ORs for the risk allele are shown for the original analysis (“Original”), in just female participants, and in just males. The p-value for the interaction between sex and the variant is also shown.

**Table S8. SuSiE Credible Set Results**

See Additional File 2

**Table S9. HLA Allele Frequencies by Study**

See Additional File 2

**Table S10. List of SNPs in the Credible Sets Excluded from eQTL Analyses**

See Additional File 2

**Table S11. eQTL Results for All Credible Set SNPs**

See Additional File 2

**Table S12. HLA Region eQTLs**

| CS | LCLs (n=398, European American) | PBMCs (n=133, African American) | NECs (n=189, African American) |
|---|---|---|---|
| Class I COA CS1/AOA CS1 | *HLA-B*<br>*MIR6891* | No eQTLs | *HLA-B* ᵞ |
| Class I COA CS2 | *CCHCR1* *<br>*AL662844.4* * | No eQTLs | No eQTLs |
| Class II COA CS1 | *HLA-DQA2* *<br>*HLA-DQB2* *<br>*HLA-DRB5* *<br>*HLA-DQB1-AS1* * | *HLA-DQA2*<br>*HLA-DQB2* | HLA-DQB2 |
| Class II COA CS2 | *HLA-DQA2*<br>*HLA-DPB2*<br>*HLA-DRB9*<br>*PSMB9*<br>*TAP2* | No eQTLs | No eQTLs |
| Class II AOA CS1 | *HLA-DQA2*<br>*HLA-DQB2*<br>*HLA-DRB6*<br>*HLA-DRB9*<br>*TAP2* | *HLA-DQA2*<br>*HLA-DQB2*<br>*HLA-DRB6* | *HLA-DQB1*<br>*HLA-DQA2*<br>*HLA-DQB2*<br>*HLA-DRB6* |
| Class II AOA CS2 | *HLA-DQA2*<br>*HLA-DQB2*<br>*HLA-DRB5*<br>*HLA-DRB6*<br>*PPT2*<br>*TAP1* | *HLA-DQA2* | *HLA-DQA1*<br>*HLA-DQA2* |

For all SNPs in each credible set (CS) and each gene within 1 Mb of the transcription start site (TSS) examined across the three datasets (lymphoblastoid cell lines [LCLs], peripheral blood mononuclear cells [PBMCs], and nasal epithelial cells [NECs], the genes with eQTLs at FDR < 0.05 are shown. n.i., no information for the SNP (Table S6). These genes were then included in the eQTL fine-mapping studies. * analyses performed in a subset of individuals who had genotypes for all SNPs in the CS. ᵞ analyses performed in a subset of individuals with genotypes from MEGA array

**Table S13. eQTL Fine-Mapping Results**

See Additional File 2

**Table S14. Amino Acid Associations with the Highest PIPs in Each Credible Set.**

| CS | HLA Locus | Pos | Ref | Alt | Frequency | p-value | OR | 95% CI | PIP | Secondary Structure |
|---|---|---|---|---|---|---|---|---|---|---|
| Class I COA CS2 | HLA-C | 11 | Ala | Ser | 0.13 | $3.12x10^{-19}$ | 0.806 | 0.768-0.844 | 0.573 | Beta Strand |
| Class II AOA CS1 | HLA-DQB1 | 55 | Lys, Pro | Arg | 0.41 | $4.50x10^{-49}$ | 0.858 | 0.841-0.876 | 0.039 | Alpha Helix |
| Class II AOA CS2 | HLA-DQA1 | 26 | Thr | Ser | 0.20 | $3.63x10^{-47}$ | 1.187 | 1.160-1.215 | 0.066 | Beta Strand |
| | | 47 | Cys | Gln | 0.20 | $3.63x10^{-47}$ | 1.187 | 1.160-1.215 | 0.066 | - |
| | | 56 | Gly,x | Arg | 0.20 | $3.63x10^{-47}$ | 1.187 | 1.160-1.215 | 0.066 | - |
| | | 76 | Ile | Val | 0.20 | $3.63x10^{-47}$ | 1.187 | 1.160-1.215 | 0.066 | Alpha Helix |
| | | 187 | Ala | Thr | 0.20 | $3.63x10^{-47}$ | 1.187 | 1.160-1.215 | 0.066 | - |

For each of the putatively causal amino acid polymorphisms in the credible sets (CSs), the position (Pos), reference (Ref) or alternative (Alt) amino acid, frequency, p-value, odds ratio (OR), 95% confidence interval (CI), posterior inclusion probability (PIP), and secondary structure are described. A dash (“-“) indicates that the variant is unlikely to reside in a functional domain.

**Table S15. Amino Acids in the Credible Sets and Their Corresponding HLA Alleles**

See Additional File 2

**Table S16. Average $r^2$ Between Childhood-Onset and Adult-Onset Asthma SNPs**

| Variant | rs2428494 (Class I COA AOA CS1) | HLA-C p.11 (Class I COA CS2) | rs28407950 (Class II COA CS1) | rs35571244 (Class II COA CS2) | rs9272346 (Class II AOA CS1) | HLA-DQA1*0301 (Class II AOA CS2) |
|---|---|---|---|---|---|---|
| rs2428494 | 1.00 | 0.0085 | 0.0154 | 0.0112 | 0.0739 | 0.0088 |
| HLA-C p.11 | | 1.00 | 0.0522 | 0.0017 | 0.0162 | 0.0055 |
| rs28407950 | | | 1.00 | 0.0097 | 0.5013 | 0.0814 |
| rs35571244 | | | | 1.00 | 0.0240 | 0.1585 |
| rs9272346 | | | | | 1.00 | 0.1622 |
| HLA-DQA1*0301 | | | | | | 1.00 |

**Table S17. Marginal vs. Conditional Association Results.**

| Variant | Marginal Association | | Conditional Association | | |
|---|---|---|---|---|---|
| **Childhood-Onset Asthma** | | | | | |
| | **p-value** | **OR [95% CI]** | **p-value** | **OR 95% CI]** | **Conditioned on** |
| rs2428292 (class I CS1) | $8.77x10^{-23}$ | 1.16 [1.12-1.19] | $5.05x10^{-13}$ | 1.11 [1.08-1.15] | rs28407950 + rs35571244 |
| HLA-C p.11 (class I CS2) | $3.12x10^{-19}$ | 0.81 [0.77-0.84] | $4.89x10^{-08}$ | 0.87 [0.83-0.92] | rs28407950 + rs35571244 |
| rs28407950 (class II CS1) | $1.37x10^{-59}$ | 0.74 [0.71-0.77] | $4.22x10^{-44}$ | 0.77 [0.74-0.80] | rs2428494 + HLA-C p.11 |
| rs35571244 (class II CS2) | $1.07x10^{-17}$ | 1.25 [1.19-1.32] | $5.59x10^{-13}$ | 1.21 [1.15-1.28] | rs2428494 + HLA-C p.11 |
| **Adult-Onset Asthma** | | | | | |
| rs2428292 (class I CS1) | $4.52x10^{-23}$ | 1.10 [1.08-1.13] | $2.03x10^{-10}$ | 1.07 [1.05-1.09] | rs9272346 + HLA-DQA1*0301 |
| rs9272346 (class II CS1) | $1.98x10^{-47}$ | 1.16 [1.14-1.19] | $1.09x10^{-34}$ | 1.14 [1.12-1.17] | rs2428494 |
| *HLA-DQA1**0301 (class II CS2) | $6.91x10^{-47}$ | 1.19 [1.16-1.21] | $1.99x10^{-41}$ | 1.18 [1.15-1.20] | rs2428494 |

P-value, odds ratio (OR), and 95% confidence interval (CI) shown for the original, marginal association and for the association when conditioning on either the class I or class II signals.

**Table S18. Sample Composition of the Replication Cohort**

| | Childhood-onset asthma | Adult-onset asthma | Controls |
|---|---|---|---|
| Sample size | 1,686 | 3666 | 56,063 |
| Age of asthma onset in years | Range: 0-11<br>Mean (SD): 6 (3) | Range: 26-65<br>Mean (SD): 43 (10) | NA |
| Female Sex | 46.1% | 64.4% | 54.6% |
| Allergic Disease (ever) | 30.5% | 26.4% | 10.3% |

**Table S19. Self-Reported Ethnic Composition of the Replication Cohort.**

| Group | COA | AOA | Ctl |
|---|---|---|---|
| White | 14 | 26 | 391 |
| British | 537 | 1063 | 16533 |
| Irish | 314 | 689 | 9651 |
| Any other white background | 395 | 830 | 13008 |
| White and Black Caribbean | 18 | 28 | 459 |
| White and Black African | 11 | 18 | 308 |
| Black or Black British | 0 | 1 | 22 |
| Caribbean | 114 | 266 | 3294 |
| African | 60 | 115 | 2826 |
| Any other Black background | 6 | 7 | 84 |
| White and Asian | 35 | 45 | 617 |
| Asian or Asian British | 0 | 5 | 30 |
| Chinese | 29 | 53 | 1311 |
| Indian | 91 | 284 | 4542 |
| Pakistani | 28 | 127 | 1352 |
| Bangladeshi | 3 | 10 | 180 |
| Any other Asian background | 31 | 99 | 1455 |

Number of individuals in each self-reported ethnic group are shown for childhood-onset asthma (COA), adult-onset asthma (AOA), and the non-asthmatic controls (Ctl).

**Table S20. Replication Meta-Analysis Results**

See Additional File 2

## Supplementary References

1. Wang G, Sarkar A, Carbonetto P, Stephens M. A simple new approach to variable selection in regression, with application to genetic fine mapping. J R Stat Soc Ser B (Statistical Methodol [Internet]. 2020 Jul 10 [cited 2020 Sep 2];82(5):1273–300. Available from: http://doi.wiley.com/10.1111/rssb.12388

2. Pirinen M, Donnelly P, Spencer CCA. Efficient computation with a linear mixed model on large-scale data sets with applications to genetic studies. Ann Appl Stat [Internet]. 2013 [cited 2020 Oct 31];7(1):369–90. Available from: https://projecteuclid.org/euclid.aoas/1365527203

3. Benner C, Spencer CCA, Havulinna AS, Salomaa V, Ripatti S, Pirinen M. FINEMAP: efficient variable selection using summary data from genome-wide association studies. Bioinformatics [Internet]. 2016 May 15 [cited 2019 Jul 27];32(10):1493–501. Available from: http://www.ncbi.nlm.nih.gov/pubmed/26773131

4. Banerjee S, Zeng L, Schunkert H, Söding J. Bayesian multiple logistic regression for case-control GWAS. PLoS Genet [Internet]. 2018 Dec 1 [cited 2020 Dec 18];14(12):e1007856. Available from: https://doi.org/10.1371/journal.pgen.1007856

5. Pividori M, Schoettler N, Nicolae DL, Ober C, Im HK. Shared and distinct genetic risk factors for childhood-onset and adult-onset asthma: genome-wide and transcriptome-wide studies. Lancet Respir Med [Internet]. 2019 Sep 26 [cited 2019 Mar 6];7(6):509–22. Available from: https://www.biorxiv.org/content/10.1101/427427v1

6. Cusanovich DA, Caliskan M, Billstrand C, Michelini K, Chavarria C, De Leon S, et al. Integrated analyses of gene expression and genetic association studies in a founder population. Hum Mol Genet [Internet]. 2016 May 15 [cited 2020 Nov 29];25(10):2104–12. Available from: https://pubmed.ncbi.nlm.nih.gov/26931462/

7. Weitkamp L, Ober C. Ancestral and recombinant 16-locus HLA haplotypes in the

Hutterites. Immunogenetics [Internet]. 1999 [cited 2019 May 2];49(6):491–7. Available from: https://www.ncbi.nlm.nih.gov/pubmed/10380692

8. Aguiar VRC, César J, Delaneau O, Dermitzakis ET, Meyer D. Expression estimation and eQTL mapping for HLA genes with a personalized pipeline. Absher DM, editor. PLOS Genet [Internet]. 2019 Apr 22 [cited 2019 Apr 27];15(4):e1008091. Available from: http://dx.plos.org/10.1371/journal.pgen.1008091

9. D'Antonio M, Reyna J, Jakubosky D, Donovan MKR, Bonder MJ, Matsui H, et al. Systematic genetic analysis of the MHC region reveals mechanistic underpinnings of HLA type associations with disease. Elife. 2019 Nov 1;8(e48476).

10. Robinson J, Halliwell JA, Hayhurst JD, Flicek P, Parham P, Marsh SGE. The IPD and IMGT/HLA database: allele variant databases. Nucleic Acids Res [Internet]. 2015 Jan [cited 2019 Mar 6];43(Database issue):D423-31. Available from: http://www.ncbi.nlm.nih.gov/pubmed/25414341

11. van de Geijn B, McVicker G, Gilad Y, Pritchard JK. WASP: allele-specific software for robust molecular quantitative trait locus discovery. Nat Methods [Internet]. 2015 Nov [cited 2019 May 5];12(11):1061–3. Available from: http://www.ncbi.nlm.nih.gov/pubmed/26366987

12. Dobin A, Davis CA, Schlesinger F, Drenkow J, Zaleski C, Jha S, et al. STAR: ultrafast universal RNA-seq aligner. Bioinformatics [Internet]. 2013 Jan 1 [cited 2019 May 4];29(1):15–21. Available from: http://www.ncbi.nlm.nih.gov/pubmed/23104886

13. Law CW, Chen Y, Shi W, Smyth GK. voom: precision weights unlock linear model analysis tools for RNA-seq read counts. Genome Biol [Internet]. 2014 Feb 3 [cited 2019 May 1];15(2):R29. Available from: http://genomebiology.biomedcentral.com/articles/10.1186/gb-2014-15-2-r29

14. Ritchie ME, Phipson B, Wu D, Hu Y, Law CW, Shi W, et al. limma powers differential expression analyses for RNA-sequencing and microarray studies. Nucleic Acids Res [Internet]. 2015 Apr 20 [cited 2019 Apr 23];43(7):e47–e47. Available from: http://academic.oup.com/nar/article/43/7/e47/2414268/limma-powers-differential-expression-analyses-for

15. Zhou X, Stephens M. Genome-wide efficient mixed-model analysis for association studies. Nat Genet [Internet]. 2012 Jun 17 [cited 2019 Jul 27];44(7):821–4. Available from: http://www.ncbi.nlm.nih.gov/pubmed/22706312

16. Gern JE, Visness CM, Gergen PJ, Wood RA, Bloomberg GR, O'Connor GT, et al. The Urban Environment and Childhood Asthma (URECA) birth cohort study: design, methods, and study population. BMC Pulm Med [Internet]. 2009 Dec 8 [cited 2019 Apr 29];9(1):17. Available from: http://www.ncbi.nlm.nih.gov/pubmed/19426496

17. Altman MC, Whalen E, Togias A, O'Connor GT, Bacharier LB, Bloomberg GR, et al. Allergen-induced activation of natural killer cells represents an early-life immune response in the development of allergic asthma. J Allergy Clin Immunol. 2018 Dec;142(6):1856–66.

18. Li H, Durbin R. Fast and accurate short read alignment with Burrows-Wheeler transform. Bioinformatics [Internet]. 2009 Jul [cited 2021 Mar 22];25(14):1754–60. Available from: https://pubmed.ncbi.nlm.nih.gov/19451168/

19. Van der Auwera GA, Carneiro MO, Hartl C, Poplin R, del Angel G, Levy-Moonshine A, et al. From fastQ data to high-confidence variant calls: The genome analysis toolkit best practices pipeline. Curr Protoc Bioinforma [Internet]. 2013 [cited 2021 Mar 22];43(SUPL.43). Available from: https://pubmed.ncbi.nlm.nih.gov/25431634/

20. Dilthey AT, Mentzer AJ, Carapito R, Cutland C, Cereb N, Madhi SA, et al. HLA*LA-HLA

typing from linearly projected graph alignments. Bioinformatics [Internet]. 2019 [cited 2020 Apr 1];35(21):4394–6. Available from: https://academic.oup.com/bioinformatics/article-abstract/35/21/4394/5426702

21. Delaneau O, Ongen H, Brown AA, Fort A, Panousis NI, Dermitzakis ET. A complete tool set for molecular QTL discovery and analysis. Nat Commun [Internet]. 2017 May 18 [cited 2020 Sep 6];8(1):1–7. Available from: www.nature.com/naturecommunications

22. Newman AM, Steen CB, Liu CL, Gentles AJ, Chaudhuri AA, Scherer F, et al. Determining cell type abundance and expression from bulk tissues with digital cytometry. Nat Biotechnol [Internet]. 2019 Jul 1 [cited 2021 Feb 17];37(7):773–82. Available from: https://doi.org/10.1038/s41587-019-0114-2

23. McKennan C, Nicolae D. Estimating and Accounting for Unobserved Covariates in High-Dimensional Correlated Data. J Am Stat Assoc [Internet]. 2020 [cited 2021 Mar 9]; Available from: https://www.tandfonline.com/doi/abs/10.1080/01621459.2020.1769635

24. Altman M, Ramratnam S, Jackson D, Presnell S, Gergen P, Bacharier L, et al. Airway Epithelium Gene Expression Endotyping of Asthma and Airway Obstruction in Urban Children [abstract]. J Allergy Clin Immunol. 2020;145(2):AB176.

25. Kent WJ, Sugnet CW, Furey TS, Roskin KM, Pringle TH, Zahler AM, et al. The Human Genome Browser at UCSC. Genome Res [Internet]. 2002 May 16 [cited 2020 Sep 3];12(6):996–1006. Available from: www.genome.org
